# Carbon–metal *versus* metal–metal synergistic mechanism of ethylene electro-oxidation *via* electrolysis of water on TM_2_N_6_ sites in graphene†

**DOI:** 10.1039/d4sc03944k

**Published:** 2024-08-02

**Authors:** Yun-Jie Chu, Chang-Yan Zhu, Chun-Guang Liu, Yun Geng, Zhong-Min Su, Min Zhang

**Affiliations:** a Institute of Functional Material Chemistry, Faculty of Chemistry, National & Local United Engineering Laboratory for Power Batteries, Northeast Normal University Changchun 130024 China mzhang@nenu.edu.cn; b Department of Chemistry, Faculty of Science, Beihua University Jilin City 132013 P. R. China liucg407@163.com; c State Key Laboratory of Supramolecular Structure and Materials, Institute of Theoretical Chemistry, College of Chemistry, Jilin University Changchun 130021 P. R. China

## Abstract

Acetaldehyde (AA) and ethylene oxide (EO) are important fine chemicals, and are also substrates with wide applications for high-value chemical products. Direct electrocatalytic oxidation of ethylene to AA and EO can avoid the untoward effects from harmful byproducts and high energy emissions. The most central intermediate state is the co-adsorption and coupling of ethylene and active oxygen intermediates (*O) at the active site(s), which is restricted by two factors: the stability of the *O intermediate generated during the electrolysis of water on the active site at a certain applied potential and pH range; and the lower kinetic energy barriers of the oxidation process based on the thermo-migration barrier from the *O intermediate to produce AA/EO. The benefit of two adjacent active atoms is more promising, since diverse adsorption and flexible catalytic sites may be provided for elementary reaction steps. Motivated by this strategy, we explored the feasibility of various homonuclear TM_2_N_6_@graphenes with dual-atomic-site catalysts (DASCs) for ethylene electro-oxidation through first-principles calculations *via* thermodynamic evaluation, analysis of the surface Pourbaix diagram, and kinetic evaluation. Two reaction mechanisms through C–TM *versus* TM–TM synergism were determined. Between them, a TM–TM mechanism on 4 TM_2_N_6_@graphenes and a C–TM mechanism on 5 TM_2_N_6_@graphenes are built. All 5 TM_2_N_6_@graphenes through the C–TM mechanism exhibit lower kinetic energy barriers for AA and EO generation than the 4 TM_2_N_6_@graphenes through the TM–TM mechanism. In particular, Pd_2_N_6_@graphene exhibits the most excellent catalytic activity, with energy barriers for generating AA and EO of only 0.02 and 0.65 eV at an applied potential of 1.77 V *vs.* RHE for the generation of an active oxygen intermediate. Electronic structure analysis indicates that the intrinsic C–TM mechanism is more advantageous than the TM–TM mechanism for ethylene electro-oxidation, and this study also provides valuable clues for further experimental exploration.

## Abstract 2

The carbon–metal synergistic mechanism is more advantageous than the metal–metal synergistic mechanism for ethylene electro-oxidation *via* the electrolysis of water on homonuclear TM_2_N_6_@graphenes.

## Graphical abstract

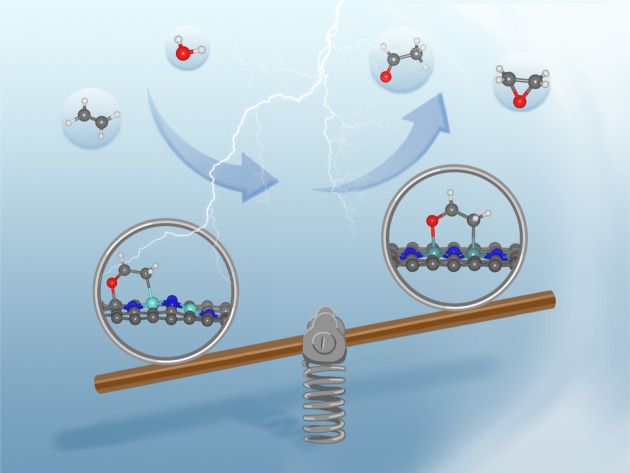

## 1 Introduction

Acetaldehyde (AA) and ethylene oxide (EO) are important fine chemicals, which can also be further converted into other successive high-value chemical products.^1,2^ Acetaldehyde, as one of the most important aldehydes, is produced in excess of 1 × 10^6^ tons per year worldwide and is widely used as a starting material for the synthesis of acetic acid, 2-ethylhexanol, acetate esters, pentaerythritol and other industrial chemicals.^2,3^ Ethylene oxide produced at about 2 × 10^6^ tons per year is widely used in the automotive industry, medicine, and other fields, and in major consumer products for antifreeze, pharmaceuticals, detergents, and plastics.^1,4^ The industrial production of AA and EO is dependent mainly on the oxidation reaction of ethylene. The main industrial process for AA production is the Wacker process, in which CuCl_2_/PdCl_2_ is utilized as a catalyst for the oxidation of ethylene in aqueous solution. Ethylene is oxidized to AA, accompanied by the reduction of Pd(ii) to Pd (0), and then Pd (0) is further re-oxidized to Pd(ii) by CuCl_2_ in a simultaneous co-catalytic cycle.^5^ Toxic and corrosive liquid byproducts are produced in this technique. Industrial production of EO from ethylene utilizes an Ag-based catalyst at 230–270 °C and 1–3 MPa to activate O_2_ and accompanied by some thermodynamic side reactions, such as the excessive oxidation of ethylene.^1,6,7^ Hence, alternative economic and environmental oxidation strategies for ethylene are desirable.

The green oxidation of alkenes through renewable electricity as a driving force is an attractive approach to achieve its chemical conversion, which has received widespread attention as it can effectively reduce carbon emissions.^8^ In the past few decades, electrochemical oxidation methods for alkenes have been reported and it has proven to be a substantial strategy.^8–13^ In 1975, Holbrook *et al.* reported that ethylene and propylene could be partially oxidized on an Ag anode, and the corresponding products were ethylene glycol and propylene oxide.^9^ In 2010, Šebera *et al.* investigating the electrochemical oxidation of ethylene on an Au electrode indicated that polycrystalline Au shows selectivity for the formation of AA.^10^ In 2020, Sargent *et al.* reported a one-step route for the electro-oxidation of ethylene to ethylene glycol under mild conditions on a gold-doped palladium catalyst as the anode with a Faraday efficiency of approximately 80% for ethylene glycol, in which the tuning of the OH binding energy is crucial for product formation.^11^ Moreover, propylene electro-oxidation has also made significant progress in recent years. In 2022, Geng *et al.* developed an efficient Ag_3_PO_4_ catalyst for the oxidation of propylene to propylene oxide starting from water electrolysis under ambient conditions, and the Ag_3_PO_4_ (100) facets achieved a product yield rate of 5.3 g_PO_ m^−2^ h^−1^ at 2.4 V *vs.* RHE.^12^ Nørskov *et al.* proved that electro-epoxidation of propylene is facile through water electrolysis to provide an oxygen source at the catalyst surface when atomic oxygen pre-exists on a catalyst surface with several weakly bound oxygens.^13^ These studies indicate great potential for the selective anodic oxidation of alkenes to AA/EO in aqueous solution under mild conditions. However, an essential understanding of the electro-oxidation process of alkenes is currently lacking, in particular a typical research case study on the direct oxidation reaction using an active oxygen intermediate (*O) generated by starting from water splitting as an oxygen source.

For the direct oxidation of ethylene, the crucial step is to introduce an oxygen atom to ethylene, which is the vital intermediate for the selective oxidation of ethylene to form AA or EO.^14–16^ It is well known that the water electrolysis process can produce an *O intermediate on the anode surface under mild conditions.^17,18^ It can be speculated that there will be a potential–pH (*U*–pH) range for *O intermediates to exist stably under electrolysis of water if the applied potential is high enough to generate *O intermediates but lower than the necessary potential value to carry out the subsequent elementary reactions or to generate other intermediates.^13,19^ The restrictive *O intermediate provides the foreground to directly oxidize ethylene at appropriate sites. More importantly, this approach avoids the use of molecular oxygen, thereby avoiding the high energy barrier of O=O cleavage and greatly reducing the hazardous complete oxidation of ethylene.

**Fig. 1:**
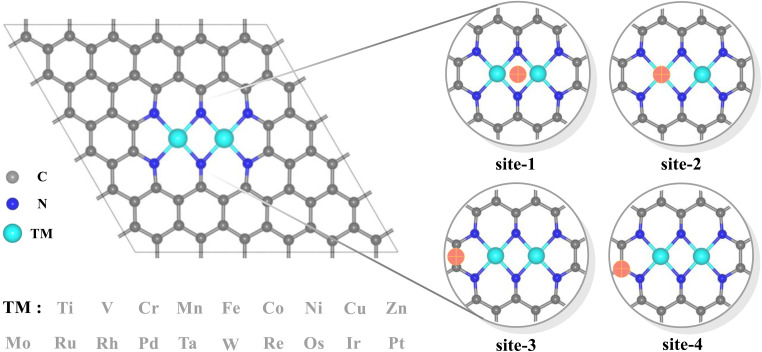
Structural model of 19 N-doped graphene-supported homonuclear transition metals, and possible adsorption sites of oxygen containing species. The metal, carbon, and nitrogen atoms, and potential adsorption sites are shown in cyan, gray, blue, and orange, respectively.

Dual-atomic-site catalysts (DASCs) by introducing an alternative active site, as an extension of single-atom catalysts (SACs), have recently drawn a surge in attention, which possessing flexible active sites when acting in isolation, offer greater probability of creating a synergistic effect, thereby producing a higher yield and achieving a higher Faraday efficiency.^20–25^ Meanwhile, the top region over carbon sites in the dipyridyl subunit also exhibits a preference for binding to oxygen-containing species besides that over two metals for several homonuclear TM_2_N_6_@graphenes.^26,27^ Hence, the TM_2_N_6_ moiety, which has been prepared experimentally^28^ with the advantage of greater possibilities for the co-adsorption of ethylene and reactive oxygen species from the two homonuclear metals and the bridge-carbon atoms adjacent to two metals, was explored in this study (as shown in Fig. 1). Furthermore, 19 homonuclear TM_2_N_6_@graphenes (Fig. 1 and S1†), with thermodynamic and electrochemical stability reported in the previous studies,^26,27,29^ were investigated here for their application to ethylene electro-oxidation. Notably, our calculated data indicates that 9 TM_2_N_6_@graphenes among them can exist in the *U*–pH range of *O intermediates, and two diverse subsequent reaction mechanisms through C–TM *versus* TM–TM synergism were determined, including 4 TM_2_N_6_@graphenes through the TM–TM mechanism and 5 TM_2_N_6_@graphenes through the C–TM mechanism. In addition, all 5 TM_2_N_6_@graphenes through the C–TM mode exhibit lower kinetic energy barriers for AA and EO generation than the 4 other TM_2_N_6_@graphenes. In particular, Pt_2_N_6_@graphene possesses the best reaction activity for the generation of AA and EO due to the lowest energy barriers for the ethylene oxidation reaction.

## 2 Computational methods

All spin-polarized DFT calculations were carried out with the Vienna *ab initio* simulation package (VASP).^30^ The Perdew–Burke–Ernzerhof (PBE) exchange correlation function of the generalized gradient approximation (GGA) was used to describe the electron interactions, and the projection augmented wave (PAW) pseudopotential was employed to treat the core electrons.^31,32^ The DFT-D3 method was adopted for the van der Waals interaction between reaction intermediates and potential catalysts.^33^ A 5 × 5 graphene supercell was employed to investigate the catalytic process and a 20 Å vacuum region was created to avoid the interaction between mirror structures. During structural optimization, the energy cutoff, and convergence criteria for the energy and force were set to 500 eV, 1 × 10^−5^ eV and 0.02 eV Å^−1^, respectively. The convergence criterion of energy was improved to 1 × 10^−7^ eV for frequency calculation. Electron spin polarization was considered in all calculations. A Monkhorst–Pack *k*-mesh of 3 × 3 × 1 was set for structural optimization and frequency calculation, and an improved 5 × 5 × 1 grid was set for electronic structure calculation. The implicit Poisson–Boltzmann solvation model^34,35^ was used to simulate the solvation effect for all reaction paths to evaluate the accuracy of the calculations, in which the dielectric constant of water was taken as 78.4, except for *ab initio* molecular dynamics (AIMD) simulations. AIMD simulations at 300 K and 500 K for 10 ps with a time step of 1 fs under NVT ensemble were made to demonstrate the stability of potential catalysts.^36^

The Gibbs free energy change (Δ*G*) of each elementary step in the OER process was obtained from the calculated hydrogen electrode (CHE) model proposed by Nørskov *et al.*^37^ The adsorption free energy of a reaction intermediate was calculated as follows:

1 Δ*G* = Δ*E*_DFT_ + Δ*E*_ZPE_ − *T*Δ*S* − *neU* − *nk*_B_*T* ln(10) × pH

where Δ*E*_DFT_ is the change in electronic energy, Δ*E*_ZPE_ is the change in zero-point energy, Δ*S* is the change in entropy, and *T* is set to 298.15 K. *n* is the number of electrons transferred in the reaction for the intermediate, *U* is the potential measured against the SHE, and *k*_B_ is the Boltzmann constant. When pH = 0:

2 Δ*G* = Δ*E*_DFT_ + Δ*E*_ZPE_ − *T*Δ*S* − *neU*

The Pourbaix diagram reveals the thermodynamically stable structures in an electrochemical system as a function of pH and applied electrode potential (*U*)^38–40^ by calculating the Δ*G* for possible intermediates in OER. Moreover, the transition states of the ethylene oxidation reactions underwent a preliminary search by the climbing-image nudged elastic band (CI-NEB) method and then by the DIMER method for further identification.^41,42^ The DIMER calculations of energy and force convergence were set to 10^−7^ eV and 0.02 eV Å^−1^. Post processing analysis of VASP was conducted with the help of the VASPKIT package.^43^

## 3 Results and discussions

### 3.1 Active oxygen intermediates (*O) and Pourbaix diagrams

Various 2D layered materials, such as graphene, graphene nitride carbon (g-C_3_N_4_) and other nitrogen-doped carbons, have emerged in recent years as supporting substrates for capturing metal dimers in experiments.^20–23,44–46^ Among these materials, nitrogen-doped graphene is regarded as one of the most promising substrates for stabilizing DASC due to its compatible ability to anchor metal, excellent catalytic performance and relatively easy preparation. In the present work, 19 homonuclear TM_2_N_6_@graphenes were chosen to explore the feasibility of ethylene electro-oxidation (Fig. 1 and S1†) and to study their intrinsic mechanism, for the following reasons: (1) this pattern of TM_2_N_6_@graphene with the five member-ring adjacent to pyridine nitrogen units has been prepared experimentally.^28^ (2) The corresponding carbon sites in the five member-ring possess the ability to adsorb oxygen containing species,^26,27^ providing more flexible adsorption sites for active oxygen and ethylene in some cases. (3) These 19 homonuclear TM_2_N_6_@graphenes possess thermodynamic and electrochemical stability, as proven in previous studies.^26,27,29^ The distance between two metal atoms is in the range of 2.23 Å (Fe_2_N_6_@graphene) to 2.67 Å (Pt_2_N_6_@graphene) in our simulated level, and most of these structures maintain planar characteristics except for a few metals with a large atomic radius that protrude upward.

According to the scheme proposed in this work, the ethylene oxidation reaction starts from ethylene and *O intermediates (generated by electrolysis of water) to produce EO or AA. The total reaction equation for ethylene oxidation is as follows:

3 *O + C_2_H_4_ → * + EO/AA

**Fig. 2:**
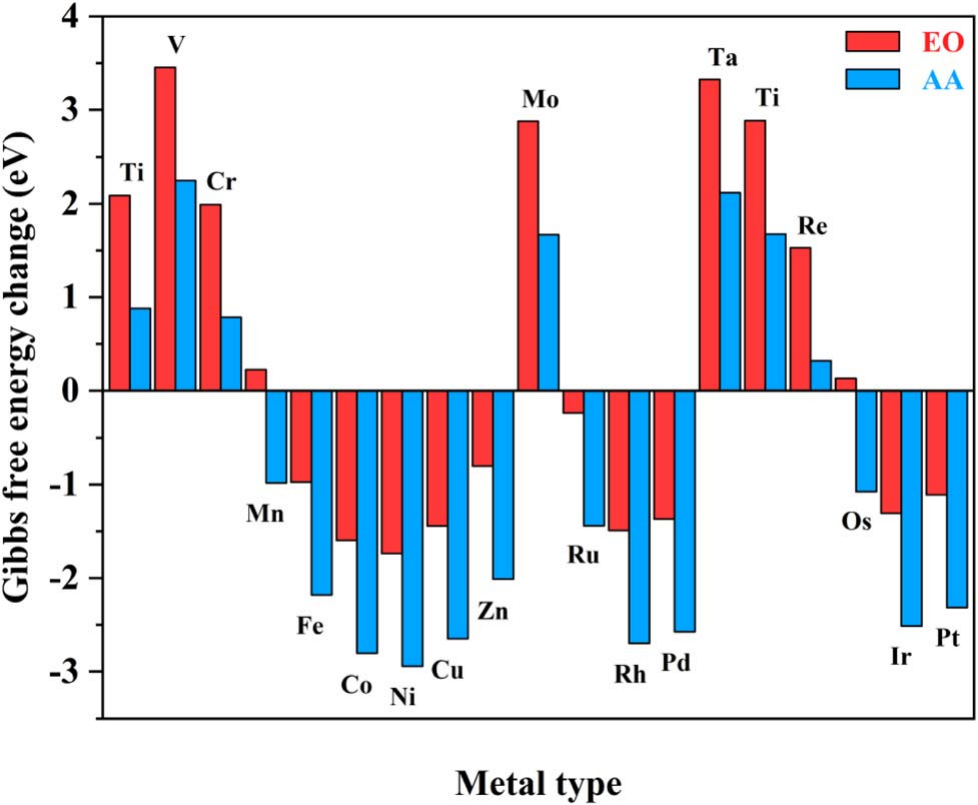
Total Gibbs free energy changes of the ethylene oxidation reaction to AA and EO initiated by *O intermediates for various TM_2_N_6_@graphenes.

Notably, the carbons of the five-member ring connected with pyridine and the metals in homonuclear TM_2_N_6_@graphene can all be utilized as potential adsorption sites for oxygen-containing species. In fact, this phenomenon has also been observed in a couple of TMN_4_ moieties in graphene, possibly due to doping-induced charge redistribution.^47^ Hence, four kinds of possible active sites were selected during electrolysis of water for oxygen-containing species, as shown in the right-hand part of Fig. 1, namely a metal–metal bridge site (site-1), a metal top site (site-2), a carbon–carbon bridge site (site-3), and a carbon top site (site-4). Firstly, for the *O intermediates of the 19 TM_2_N_6_@graphenes, the structures of the four potential sites are shown in Fig. S2–S5† and the relative energies are listed in Table S1.† The total thermodynamic Gibbs free energy changes (Δ*G*) of ethylene oxidation initiated by stable *O intermediates to AA and EO is shown in Fig. 2, and the relevant data are also listed in Table S2.† The ethylene oxidation reaction requires effective adsorption of an ethylene molecule on the *O intermediate, which is an exothermic or slightly endothermic process, followed by the formation of AA or EO on the catalyst surface after undergoing one corresponding transition state, and subsequent desorption. The transition-state energy barriers must in general be higher than the Gibbs free energies of the initial to final states. If the Gibbs free energies of the final states are higher, the barrier energies of their transition states must be even higher. As shown in Fig. 2 and Table S2,† there are 12 TM_2_N_6_@graphenes (TM = Mn, Fe, Co, Ni, Cu, Zn, Ru, Rh, Pd, Os, Ir, and Pt) which almost possess negative Δ*G* of the final states for generating AA and EO simultaneously, except for the slightly endothermic generation of EO on Mn_2_@graphenes (0.23 eV) and Os_2_N_6_@graphenes (0.13 eV). Hence, these 12 TM_2_N_6_@graphenes were further investigated in detail for ethylene electro-oxidation.

**Fig. 3:**
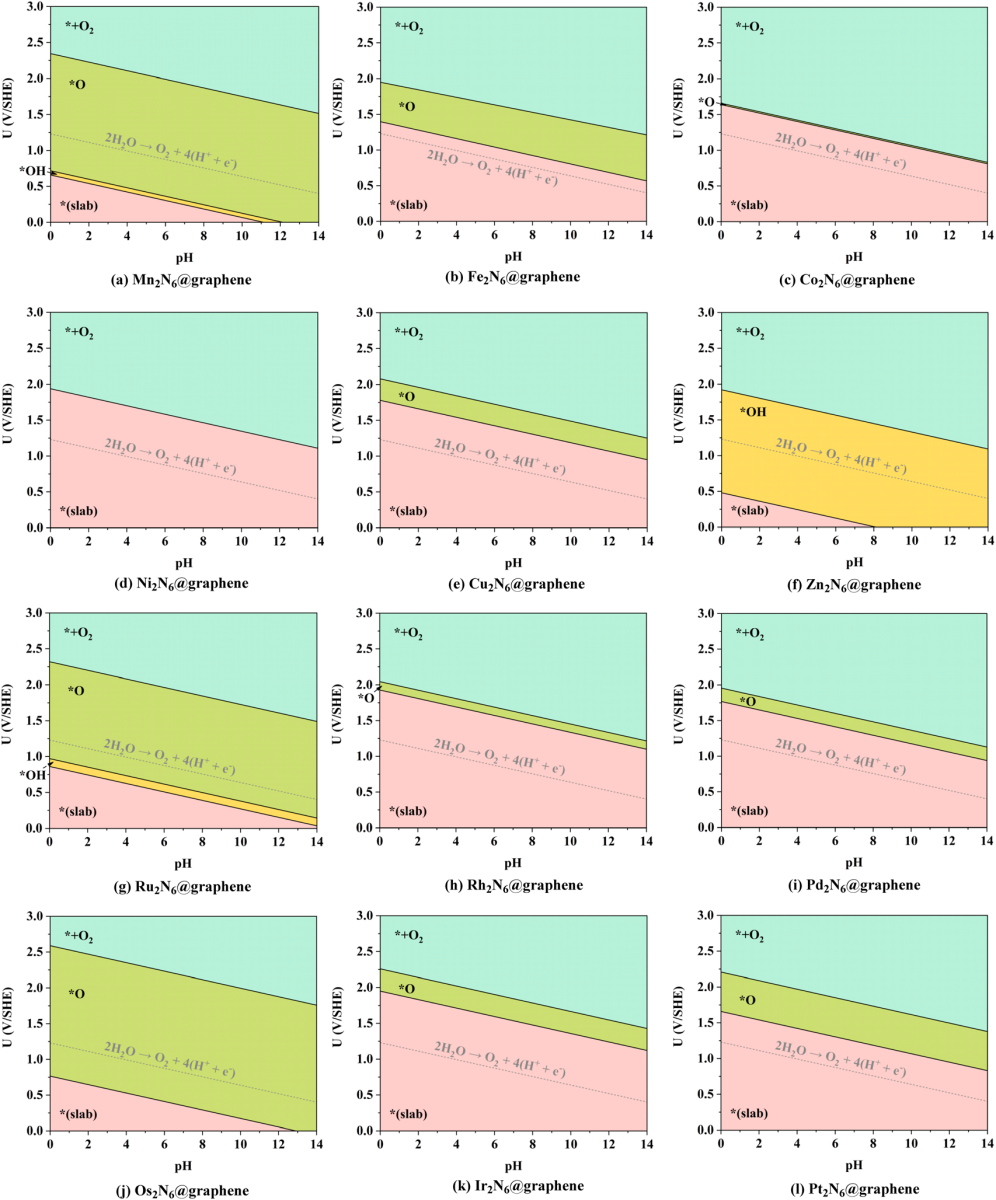
Pourbaix diagrams on the surfaces of 12 TM_2_N_6_@graphenes under the given applied potential value of 0–3 V against the SHE and pH scale of 0–14 at their most stable adsorption sites. Pink, yellow, green, and cyan represent the *U*–pH ranges in which the intermediates of *(slab), *OH, *O, and *+O_2_ can be stabilized, respectively.

As mentioned above, the source of active oxygen species, namely the *O intermediates, is assumed to be generated by the electrolysis of water, thus avoiding the traditional drawbacks caused by O=O bond breakage. Therefore, a wide potential–pH (*U*–pH) range is optimal and desirable to stabilize the *O intermediate in the electrochemical process. Specifically, only *O intermediates can exist, *i.e.* ΔG_*O_ < 0, and ΔG_*O_ < ΔG_*OOH_, which means that the generation of *O intermediates cannot be overwhelmed by the generation of *OOH during OER under the certain applied potential and pH conditions. Here, possible adsorption intermediates were considered and the optimized structures are shown in Fig. S2–S10,† and the Pourbaix diagrams of these 12 TM_2_N_6_@graphenes were constructed (Fig. 3) with the most thermodynamically stable intermediates during OER in our calculations. As shown in Fig. 3, the thermodynamically stable range for the 12 TM_2_N_6_@graphene surfaces during OER was explored under the given applied potential values of 0–3 V against the SHE and a pH of 0–14. Firstly, for Mn_2_-, Ru_2_-, and Os_2_N_6_@graphenes (Fig. 3a, g, and j), there exists a very wide range to stabilize the *O intermediate, and the lowest applied potential for generating the *O intermediates are only 0.72, 0.97, and 0.76 V, respectively, at pH = 0. All of Fe_2_-, Cu_2_-, Rh_2_-, Ir_2_-, and Pt_2_N_6_@graphenes (Fig. 3b, e, h, and k), have a moderate *U*–pH range to stabilize the *O intermediate, and the applied potentials for generating *O intermediates of these surfaces are below 2.0 V, lower than the reaction of propylene electro-epoxidation at 2.4 V *vs.* RHE on the Ag_3_PO_4_ (100) facets.^12^ Secondly, for Co_2_N_6_@graphene (Fig. 3c), there is only a very narrow *U*–pH range with *O intermediate, and its area ratio is only 0.67% for the given range. However, for completeness of the data, we also conducted the following studies on Co_2_N_6_@graphene. Thirdly, for Ni_2_- and Zn_2_N_6_@graphenes (Fig. 3d and f), there is no *U*–pH range that can stabilize the *O intermediate. The applied potential necessary to generate the *OH intermediate (from * → *OH, pH = 0, *U* = 1.94 V, or pH = 14, *U* = 1.11 V) can directly complete the OER process on Ni_2_N_6_@graphene. The applied potential for generating the *O intermediate (from *O → *OH, pH = 0, *U* = 1.92 V, or pH = 14, *U* = 1.09 V) are higher than the applied potential for generating the *OOH intermediate from *O (from *O → *OOH, pH = 0, *U* = 1.65 V, or pH = 14, *U* = 0.82 V) on Zn_2_N_6_@graphene. Therefore, although both Ni_2_-, and Zn_2_N_6_@graphenes exhibit good OER catalytic performance with applied overpotentials of 0.71 and 0.69 V at pH = 0, they are not suitable for the ethylene electro-oxidation reaction. Meanwhile, it should be noted that surface Pourbaix diagrams have been constructed for all 12 TM_2_N_6_@graphene surfaces, on which the *OH intermediates can be adsorbed on three potential sites except for site-3, and the relevant structures and the relative energies are shown in Fig. S6–S8† and listed in Table S3.† The *OOH and *OO intermediates cannot be adsorbed around the C sites, and the most stable structures are shown in Fig. S9 and S10.† (It should be noted that it is a desorption thermochemical process from *OO to *+O_2_, and the Δ*G* of O_2_ desorption from TM_2_N_6_@graphenes are 0.71 eV from Mn_2_-, 0.05 eV from Fe_2_-, −0.35 eV from Co_2_-, −0.42 eV from Ni_2_-, −0.36 eV from Cu_2_-, −0.02 eV from Zn_2_-, 0.24 eV Ru_2_-, −0.50 eV from Rh_2_-, −0.18 eV from Pd_2_-, 0.21 eV from Os_2_-, −0.46 eV from Ir_2_-, and −0.11 eV from Pt_2_N_6_@graphenes, respectively), and there are no *U*–pH ranges of *OOH intermediates for the 12 TM_2_N_6_@graphenes. In summary, 10 potential TM_2_N_6_@graphenes are eligible for ethylene electro-oxidation based on their Pourbaix diagrams.

### 3.2 Ethylene oxidation during OER

For the 10 TM_2_N_6_@graphenes to be further studied in detail, the most stable *O intermediates on 4 surfaces are the O adsorption on the metal sites (site-1 for Fe_2_N_6_@graphene, and site-2 on Mn_2_-, Ru_2_-, and Os_2_N_6_@graphenes), and those on 6 surfaces are the C adsorption of O on C sites (site-4 on Co_2_-, Rh_2_-, Ir_2_-, Pd_2_-, Pt_2_- and Cu_2_N_6_@graphenes). Therefore, the difference in their most stable O adsorption sites on various TM_2_N_6_@graphene surfaces leads to the decoupling difference of C–O bonding and TM–O bonding and the subsequent coupling oxidation progress between O and ethylene.

**Fig. 4:**
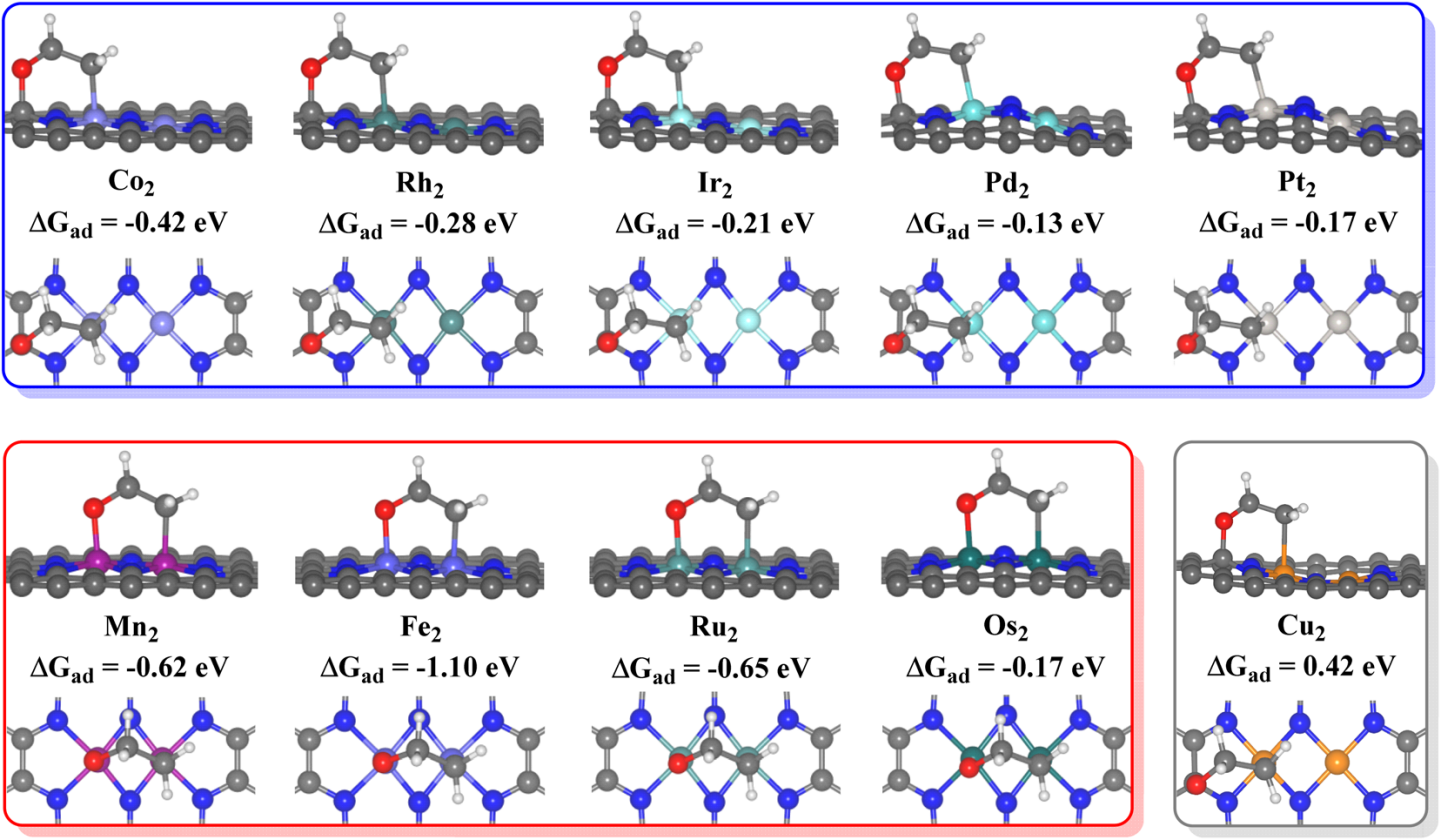
Side and top views of adsorption structures of ethylene on the *O surface of 10 TM_2_N_6_@graphenes, and their adsorption energies (Δ*G*_ad_) in eV.

The ethylene oxidation reaction also requires the effective adsorption of an ethylene molecule on the adjacent sites with the *O intermediate as mentioned above, so the stable configurations of ethylene adsorption on the *O intermediates of the 10 TM_2_N_6_@graphenes are approached and shown in Fig. 4. Therefore, the ethylene oxidation was divided into two mechanisms for the formation of AA or EO in this study through different O adsorption sites, namely the metal–metal (TM–TM) synergistic mechanism and the carbon–metal (C–TM) synergistic mechanism, which are very similar to the oxametallacycle intermediate (OMME).^1,48^ Notably, the calculations show that the O moves from site 1 to site 2 on Fe_2_N_6_@graphene while stabilizing the adsorption structure of ethylene. The calculated adsorption energies (Δ*G*_ad_) of ethylene on *O intermediates through the TM–TM mode are −0.62, −1.10, −0.65, and −0.17 eV for Mn_2_-, Fe_2_-, Ru_2_-, and Os_2_N_6_@graphenes, respectively. The corresponding Δ*G*_ad_ through the C–TM mode are −0.42. −0.28, −0.21, −0.13, and −0.17 eV for Co_2_-, Rh_2_-, Ir_2_-, Pd_2_-, Pt_2_N_6_@graphenes, respectively. Only the corresponding Δ*G*_ad_ is an endothermic process of 0.42 eV through the C–TM mode for Cu_2_N_6_@graphene, which may be a very weak physical adsorption mode and may not possess a comparable potential for ethylene electro-oxidation. Hence, the focus will be on the two kinds of ethylene electro-oxidation on the other 9 TM_2_N_6_@graphenes through C–TM and TM–TM modes in the following part.

**Fig. 5:**
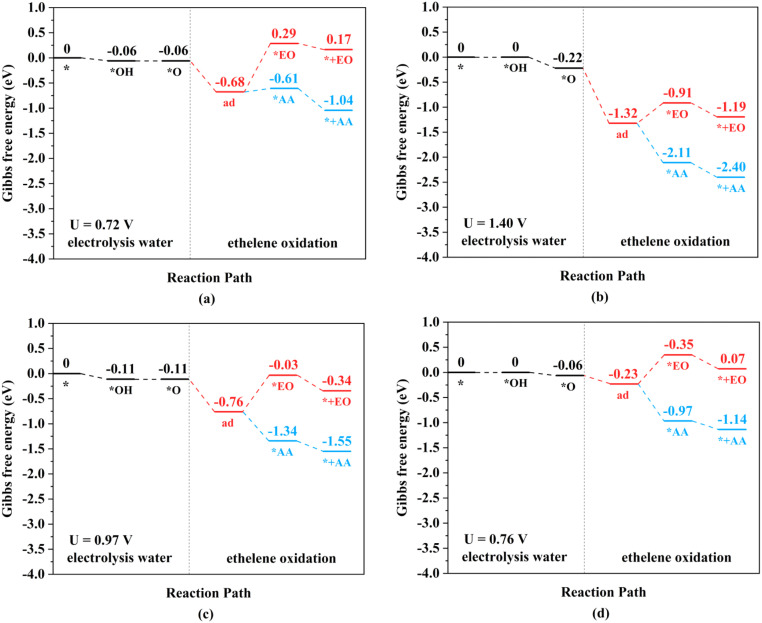
Gibbs free energy change diagrams for ethylene electro-oxidation on (a) Mn_2_-, (b) Fe_2_-, (c) Ru_2_-, and (d) Os_2_N_6_@graphenes at the applied potential at which the *O intermediates can generated at pH = 0.

**Fig. 6:**
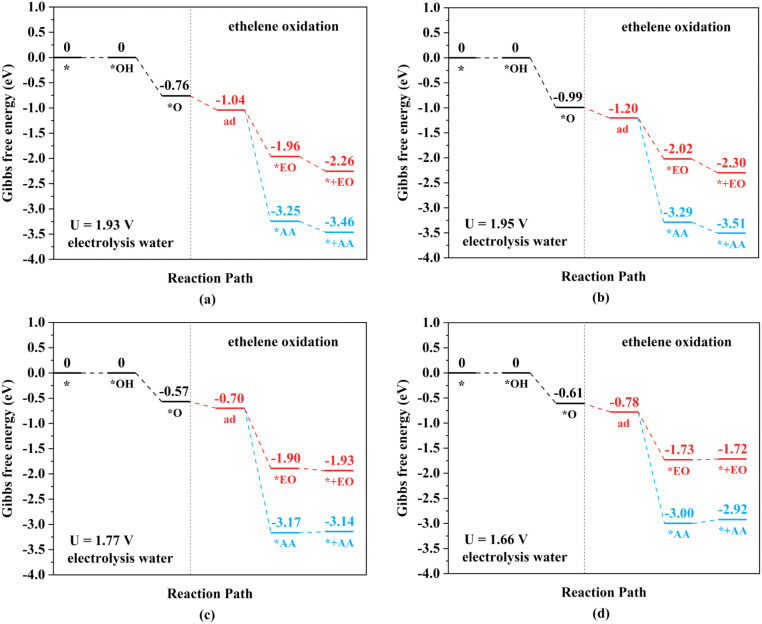
Gibbs free energy change diagrams for ethylene electro-oxidation on (a) Rh_2_-, (b) Ir_2_-, (c) Pd_2_-, and (d) Pt_2_N_6_@graphenes at the lowest applied potential to generate *O intermediates at pH = 0.

The ethylene electro-oxidation reaction can be divided into two continuous parts: a water electrolysis process and a thermochemical ethylene oxidation process. The first half is the electrochemical process of water electrolysis at the lowest applied potential to generate the *O intermediates, while the second half is the thermochemical process for ethylene oxidation to AA and EO, including ethylene adsorption adjacent to the *O intermediates on TM_2_N_6_@graphenes, the production of *AA and *EO, and then their final desorption. The thermodynamic steps and the free energy changes (Δ*G*) of electrochemistry and thermochemistry on 9 selected TM_2_N_6_@graphenes are shown in Fig. 5, 6 and S11.† The adsorption states of ethylene on the *O intermediate towards AA (*AA as shown in Fig. S12†) are exothermic processes through the TM–TM mode for Fe_2_-, Ru_2_-, and Os_2_N_6_@graphenes, except for a slightly endothermic process for Mn_2_N_6_@graphenes. The following desorptions of AA from the catalyst surface are all exothermic. The adsorption states of EO (*EO as shown in Fig. S13†) are all endothermic processes for the 4 TM_2_N_6_@graphenes, and then the corresponding desorptions of EO are exothermic. In contrast, the adsorption states of ethylene towards *AA or *EO are all obviously exothermic processes through the C–TM mode of Rh_2_-, Ir_2_, Pd_2_-, Pt_2_-, and Co_2_N_6_@graphenes. Subsequently, the desorption of AA from Pd_2_-, Pt_2_- and Co_2_N_6_@graphenes and the desorption of EO from Pt_2_-, and Co_2_N_6_@graphene are slightly endothermic, but the desorption of other products from TM_2_N_6_@graphenes is exothermic.

**Fig. 7:**
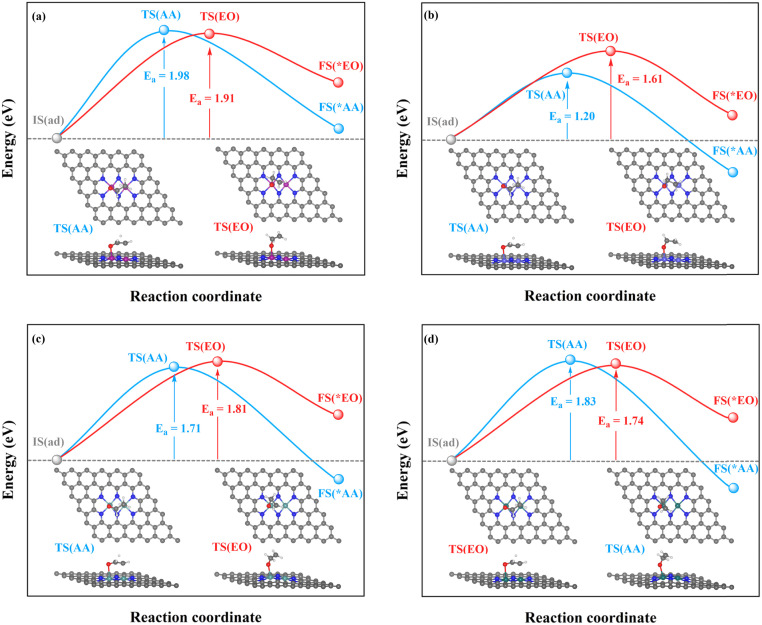
Kinetic energy barriers and transition-state structures of AA and EO generation through the TM–TM mechanism on (a) Mn_2_-, (b) Fe_2_-, (c) Ru_2_-, and (d) Os_2_N_6_@graphenes.

**Fig. 8:**
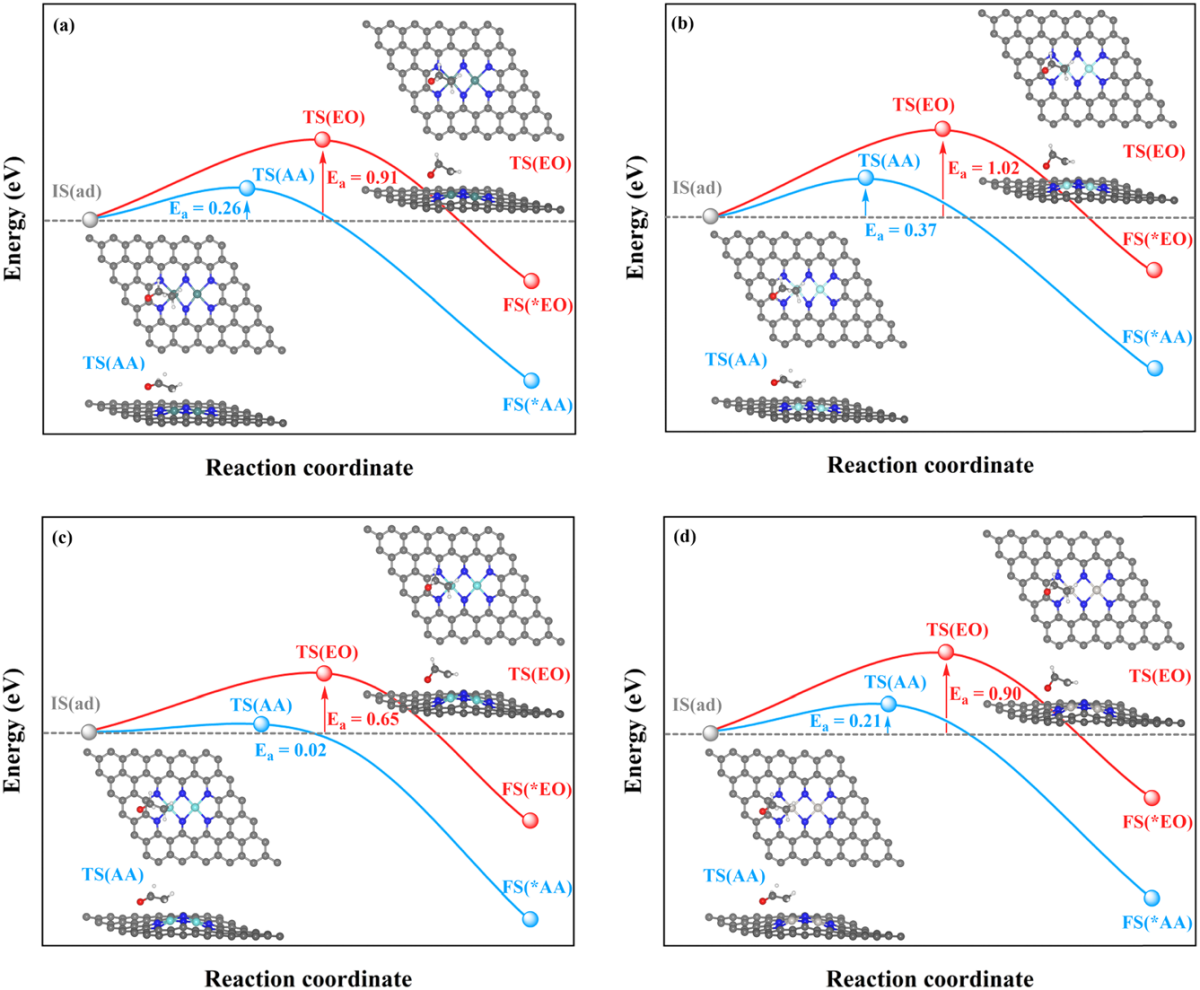
Kinetic energy barriers and transition-state structures of AA and EO generation through the C–TM synergistic mechanism on (a) Rh_2_-, (b) Ir_2_-, (c) Pd_2_-, and (d) Pt_2_N_6_@graphenes.

The TM–TM mechanism exhibits a lower applied potential for the generation of *O intermediates during their electrochemical process than in the previous discussion, but the C–TM mechanism is thermodynamically more favorable for a thermochemical process. More importantly, their kinetic energy barriers of ethylene oxidation are decisive for catalyst activity and final product selectivity, since they cannot be controlled by the applied potential. The kinetic energy barriers to AA or EO were further investigated to evaluate the catalytic activity and selectivity, which are given in Fig. 7, 8 and S14.†

Through the TM–TM mode the barriers for the formation of EO (1.91 and 1.74 eV) are lower than those of AA (1.98 and 1.83 eV) on Mn_2_- and Os_2_N_6_@graphenes, and the barriers for the formation of AA (1.20 and 1.71 eV) are lower than those of EO (1.61 and 1.81 eV) on Fe_2_- and Ru_2_N_6_@graphenes. Through the C–TM mode the barriers of AA (0.26 eV on Rh_2_-, 0.37 eV on Ir_2_-, 0.02 eV on Pd_2_-, and 0.21 eV on Pt_2_-, and 0.36 eV on Co_2_N_6_@graphenes, respectively) are lower than those of EO (0.91 eV on Rh_2_-, 1.02 eV on Ir_2_-, 0.65 eV on Pd_2_-, and 0.90 eV on Pt_2_-, and 1.06 eV on Co_2_N_6_@graphenes, respectively). Hence, these data indicate that the thermochemical ethylene oxidation process through the C–TM synergistic mechanism is superior to that through the TM–TM synergistic mechanism. In particular, both AA and EO generation on Pd_2_N_6_@graphene present very low kinetic energy barriers.

### 3.3 The origin of TM–TM and C–TM synergistic mechanisms

The intrinsic nature of ethylene electro-oxidation in our scheme is the stable existence of *O intermediates on active sites of TM_2_N_6_@graphene surfaces in a certain *U*–pH range during the electrolysis water (Fig. 3), the formation of the *OC_2_H_4_ intermediate through the TM–TM and C–TM synergistic mechanisms after the introduction of ethylene, and the execution of ethylene oxidation reactions towards *AA or *EO. During the whole process, it is crucial to understand *O stability on TM sites and C sites, which determines the following TM–TM and C–TM synergistic mechanisms on TM_2_N_6_@graphene surfaces.

On the other hand, it is well known that the d-band center of a transition metal is an effective descriptor related to the adsorption behavior and the corresponding energy between the adsorbate and the substrate.^49,50^ The projected density of states (PDOS) of the d orbitals of the two metals in the 9 promising TM_2_N_6_@graphenes involved in the two reaction mechanisms are shown in Fig. S15.† In general, compared to the d orbital distributions of the 4 TM_2_N_6_@graphenes through the TM–TM mode (Fig. S15a–d†), those of the 5 TM_2_N_6_@graphenes (Fig. S15e–i†) through the C–TM mode are further away from the Fermi level. Therefore, the distinctive *O stability on different TM_2_N_6_@graphene surfaces can be attributed to their diversity of d orbital distributions in the metals, which is consistent with previous reports.^26,27^

**Fig. 9:**
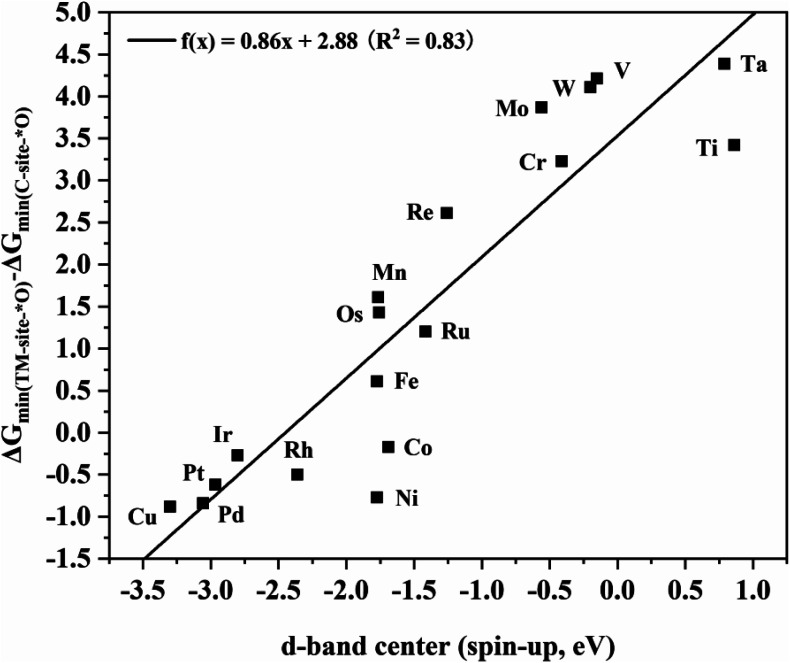
Linear relationship between the d-band center of metals in TM_2_N_6_@graphenes and the energy difference of the most stable O adsorption sites between the metal and C sites.

To further explore the intrinsic correlation between the distribution of d orbitals and the adsorption energy of O at different sites, the relationship between the d-band center of metals in various TM_2_N_6_@graphenes and the energy difference of the most stable adsorption structure of O on the metal and C sites is shown in Fig. 9. There exists an approximately linear correlation between these two values on various TM_2_N_6_@graphenes except on Zn_2_N_6_@graphene, where *f*(*x*) = 0.86*x* + 2.88, *R*^2^ = 0.83. Moreover, OH is also adsorbed on these sites, and the relationship between the d-band center of the metals in the 9 promising TM_2_N_6_@graphenes involved in the two mechanisms in this study and the energy difference of the most stable adsorption structure of OH on these sites is another approximately linear correlation, as shown in Fig. S16,† where *f*(*x*) = 0.83*x* + 2.35, *R*^2^ = 0.76. This is close to the trend shown in Fig. 9. In fact, the narrow difference in energy between the occupied higher d-band center (closer to the Fermi level) of the metal and the unoccupied higher anti-bonding orbitals of the adsorbed molecule (closer to the Fermi level) will lead to stronger interaction and higher energy release after molecule adsorption.^51^ In addition, some of the electrons transfer from the metal to the O atom when the O atom is adsorbed on the metal, so the d-band center being much closer to the Fermi level results in many more electrons transferring to the O atom, which further stabilizes the adsorption of the O atom to the metal. These two reasons can explain why the metal sites of TM_2_N_6_@graphenes studied in this work exhibit lower competitive adsorption ability than C sites for active oxygen species as the d-band center of the metals decreases. As a result, a higher the d-band center results in stronger ability to bind an O atom. Moreover, the O atom must be separated from the binding site to generate AA or EO for the ethylene oxidation process. Therefore, the C–TM synergistic mechanism exhibits lower kinetic energy barriers than the TM–TM mechanism. These are the trends which correspond to the overall catalytic activity in Fig. 9 and S16,† where TM_2_N_6_@graphenes through the C–TM mode locate at the lower-left part around the lines, and TM_2_N_6_@graphenes through the TM–TM mode locate at the middle and upper-right part around the lines. That is, these data strongly support the proposition that ethylene electro-oxidation on TM_2_N_6_@graphenes through the C–TM synergistic mechanism possess higher catalytic activity and lower kinetic energy barrier than the process through the TM–TM synergistic mechanism. It is hoped that our findings on the C–TM *versus* the TM–TM synergistic mechanism can provide potential help to explore and screen other promising electro-catalysts.

Finally, the 9 promising TM_2_N_6_@graphene catalysts were studied by AIMD simulations at 300 K and 500 K for 10 ps, as shown in Fig. S17 and S18.† The frameworks of these catalysts were well maintained in their original configurations with the TMN_6_ moiety in the final snapshots of the AIMD simulations. These results further demonstrate their outstanding thermal stability at high temperature.

## 4 Conclusions

In this work, the feasibility of 19 homonuclear TM_2_N_6_@graphenes with dual-atomic-site catalysts for ethylene electro-oxidation to AA and EO through active oxygen intermediates generated by electrolysis of water was investigated in detail. Most of all, a reasonable strategy for generating an active oxygen intermediate is proposed that can exist stably during the electrolysis of water, and further oxidation of ethylene, thereby avoiding the potential drawbacks of traditional industrial processes. Fine promising TM_2_N_6_@graphenes including four through the metal–metal synergistic mechanism and five through the carbon–metal synergistic mechanism were selected according to the adsorption sites of oxygen-containing species, Pourbaix diagram analysis on their surfaces, the lowest applied potential for *O intermediate generation, and their thermodynamic and kinetic evaluation. For the TM–TM mode, the kinetic energy barriers are superior for EO formation on Fe_2_-, and Os_2_N_6_@graphenes, while they are more favorable for AA formation on Mn_2_-, and Ru_2_N_6_@graphenes. For the C–TM mode, all Rh_2_-, Ir_2_-, Pd_2_-, Pt_2_-, and Co_2_N_6_@graphenes possess lower formation barriers to both AA and EO. In particular, Pd_2_N_6_@graphenes have very low kinetic energy barriers of 0.02 and 0.65 eV for both AA and EO formation at an applied potential of 1.77 V *vs.* RHE for the generation of an *O intermediate. Electronic structure analysis indicates that there exists an approximately linear correlation between the d-band center of metals in TM_2_N_6_@graphenes and the energy difference of the most stable adsorption structure of O on the metal and C sites, where *f*(*x*) = 0.86*x* + 2.88, *R*^2^ = 0.83. The data reflect the intrinsic variance caused by the ability of the metal to adsorb the O atom, quantified by the d-band center of the metals decreasing, where TM_2_N_6_@graphenes through the C–TM mode locate at the lower-left part around the lines. Therefore, we believe that this work provides valuable clues for further experimental exploration and will stimulate more research to further explore the electro-oxidation of hydrocarbons.

## Author contributions

Y. J. C. and M. Z. designed the research. Y. J. C. and C. Y. Z. demonstrated the initial idea and collected all the data. Y. J. C. wrote the paper. C. G. L., Y. G., Z. M. S. and M. Z. revised the paper and all authors commented on it.

## Conflicts of interest

There are no conflicts to declare.

## Supplementary Material

SC-015-D4SC03944K-s001

## Data Availability

All the data supporting this article have been included in the main text and the ESI.†
