# Supplementary material for: Carbon–metal versus metal–metal synergistic mechanism of ethylene electro-oxidation via electrolysis of water on TM2N6 sites in graphene
Source: Chem Sci. 2024 Aug 2;15(34):13976–86. doi: 10.1039/d4sc03944k (PMC11320337; doi:10.1039/d4sc03944k)
Supplement: SC-015-D4SC03944K-s001 [file SC-015-D4SC03944K-s001.pdf]

Supplementary Information (SI) for Chemical Science.
This journal is © The Royal Society of Chemistry 2024

Supporting Information for

Carbon-metal versus Metal-metal Synergistic Mechanism of Ethylene Electro-oxidation via Electrolysis Water on TM_2N_6 sites in Graphene

Yun-Jie Chu,[a] Chang-Yan Zhu,[a] Chun-Guang Liu,[b*] Yun Geng,[a] Zhong-Min Su[c] and Min Zhang[a*]

[a]Institute of Functional Material Chemistry, Faculty of Chemistry, National & Local United Engineering Laboratory for Power Batteries, Northeast Normal University, Changchun 130024, China;

[b]Department of Chemistry, Faculty of Science, Beihua University, Jilin City 132013, P. R. China

[c]State Key Laboratory of Supramolecular Structure and Materials, Institute of Theoretical Chemistry, College of Chemistry, Jilin University, Changchun, 130021 P. R. China

Email: liucg407@163.com; mzhang@nenu.edu.cn

Table of contents

(Total Number of Pages: 24, Total Number of Figures: 18, Total Number of Tables: 3)

**Table S1.** Free energy difference (ΔG, eV) of O atom adsorbed at difference sites for 19 TM_2N_6@graphenes, the "0 eV" represents the most stable O adsorption site, and "-" represents no corresponding configurations obtained during their optimization.	Page S4
**Table S2.** Total Gibbs free energy changes of ethylene oxidation reaction initiated by stable *O intermediates to AA and EO on various TM_2N_6@graphenes.	Page S5
**Table S3.** Gibbs free energy difference (ΔG, eV) of OH adsorbed at difference sites on 12 TM_2N_6@graphenes, the "0 eV" represents the most stable OH adsorption site, and "-" represents no corresponding configurations obtained during their optimization.	Page S6
**Fig. S1** Top and side views of various homonuclear TM_2N_6@graphene DMACs with distances between two metal atoms in angstroms (Å).	Page S7
**Fig. S2** Top and side views of O atom absorbed at the **site-1** on various TM_2N_6@graphenes.	Page S8
**Fig. S3** Top and side views of O atom absorbed at **site-2** on various TM_2N_6@graphenes.	Page S9
**Fig. S4** Top and side views of O atom absorbed at **site-3** on various TM_2N_6@graphenes.	Page S10
**Fig. S5** Top and side views of O atom absorbed at **site-4** on various TM_2N_6@graphenes.	Page S11
**Fig. S6** Top and side views of OH absorbed at **site-1** on various TM_2N_6@graphenes.	Page S12
**Fig. S7** Top and side views of OH absorbed at **site-2** on various TM_2N_6@graphenes.	Page S13
**Fig. S8** Top and side views of OH absorbed at **site-4** on various TM_2N_6@graphenes.	Page S14
**Fig. S9** Top and side views of OOH absorbed on various TM_2N_6@graphenes.	Page S15
**Fig. S10** Top and side views of OO absorbed on various TM_2N_6@graphenes.	Page S16
**Fig. S11** Gibbs free energy change diagrams for ethylene electro-oxidation on Co_2N_6@graphenes at the lowest applied potential to generate *O intermediates at pH=0.	Page S17
**Fig. S12** Top and side views of acetaldehyde absorbed on various TM_2N_6@graphenes.	Page S18
**Fig. S13** Top and side views of ethylene oxide absorbed on various TM_2N_6@graphenes.	Page S19
**Fig. S14** Kinetic energy barriers and transition state structures of AA and EO generation through C-TM mechanism on Co_2N_6@graphenes.	Page S20

**Fig. S15** Projected density of states (PDOS) of the d orbitals of the two metals in TM_2N_6@graphenes.	Page S21
**Fig. 16** Linear relationship between d-band center of metals in TM_2N_6@graphenes and energy difference of the most stable OH adsorption sites between metal and C sites.	Page S22
**Fig. S17** Evolution of the total energy of 9 TM_2N_6@graphene at 300 K, and the total times duration 10 ps. Snapshots of structures at the end of AIMD simulations are also shown.	Page S23
**Fig. S18** Evolution of the total energy of 9 TM_2N_6@graphene at 500 K, and the total times duration 10 ps. Snapshots of structures at the end of AIMD simulations are also shown.	Page S24

Table S1. Free energy difference (ΔG, eV) of O atom adsorbed at difference sites for 19 TM_2N_6@graphenes, the "0 eV" represents the most stable O adsorption site, and "-" represents no corresponding configurations obtained during their optimization.

metal	site-1	site-2	site-3	site-4
TiTi	0	—	3.42	—
VV	0.17	0	4.21	—
CrCr	0.77	0	3.28	3.23
MnMn	0.43	0	1.74	1.61
FeFe	0	0.03	0.63	0.61
CoCo	0.17	0.17	0.17	0
NiNi	0.90	0.77	0.13	0
CuCu	0.93	0.88	0.09	0
ZnZn	0	—	0.43	0.39
MoMo	0.76	0	3.87	—
RuRu	0.44	0	1.23	1.20
RhRh	1.11	0.50	0.23	0
PdPd	1.20	0.84	0.09	0
TaTa	0	0.18	4.39	—
WW	0.73	0	4.11	—
ReRe	0.92	0	2.65	2.61
OsOs	1.56	0	1.48	1.43
IrIr	—	0.27	0.30	0
PtPt	1.44	0.62	0.20	0

Table S2. Total Gibbs free energy changes of ethylene oxidation reaction initiated by stable *O intermediates to AA and EO on various TM_2N_6@graphenes.

metal type	Ti	V	Cr	Mn	Fe
ΔG_{AA} (eV)	0.88	2.25	0.78	-0.98	-2.18
ΔG_{EO} (eV)	2.09	3.46	1.99	0.23	-0.97
metal type	Co	Ni	Cu	Zn	Mo
ΔG_{AA} (eV)	-2.80	-2.95	-2.65	-2.01	1.67
ΔG_{EO} (eV)	-1.60	-1.74	-1.44	-0.80	2.88
metal type	Ru	Rh	Pd	Ta	W
ΔG_{AA} (eV)	-1.44	-1.49	-2.58	2.12	1.68
ΔG_{EO} (eV)	-0.23	-2.70	-1.37	3.33	2.83
metal type	Re	Os	Ir	Pt	
ΔG_{AA} (eV)	0.32	-1.08	-2.51	-2.32	
ΔG_{EO} (eV)	1.53	0.13	-1.31	-1.11	

Table S3. Gibbs free energy difference (ΔG, eV) of OH adsorbed at difference sites on 12 TM_2N_6@graphenes, the "0 eV" represents the most stable OH adsorption site, and "-" represents no corresponding configurations obtained during their optimization.

metal	site-1	site-2	site-3	site-4
MnMn	—	0	—	1.30
FeFe	0.09	0	—	0.66
CoCo	—	0	—	0.48
NiNi	—	0	—	0.19
CuCu	0.02	0.02	—	0
ZnZn	0.09	0	—	1.25
RuRu	1.12	0	—	1.21
RhRh	—	0	—	0.18
PdPd	—	0.06	—	0
OsOs	1.54	0	—	1.29
IrIr	—	0	—	0.07
PtPt	—	0.16	—	0

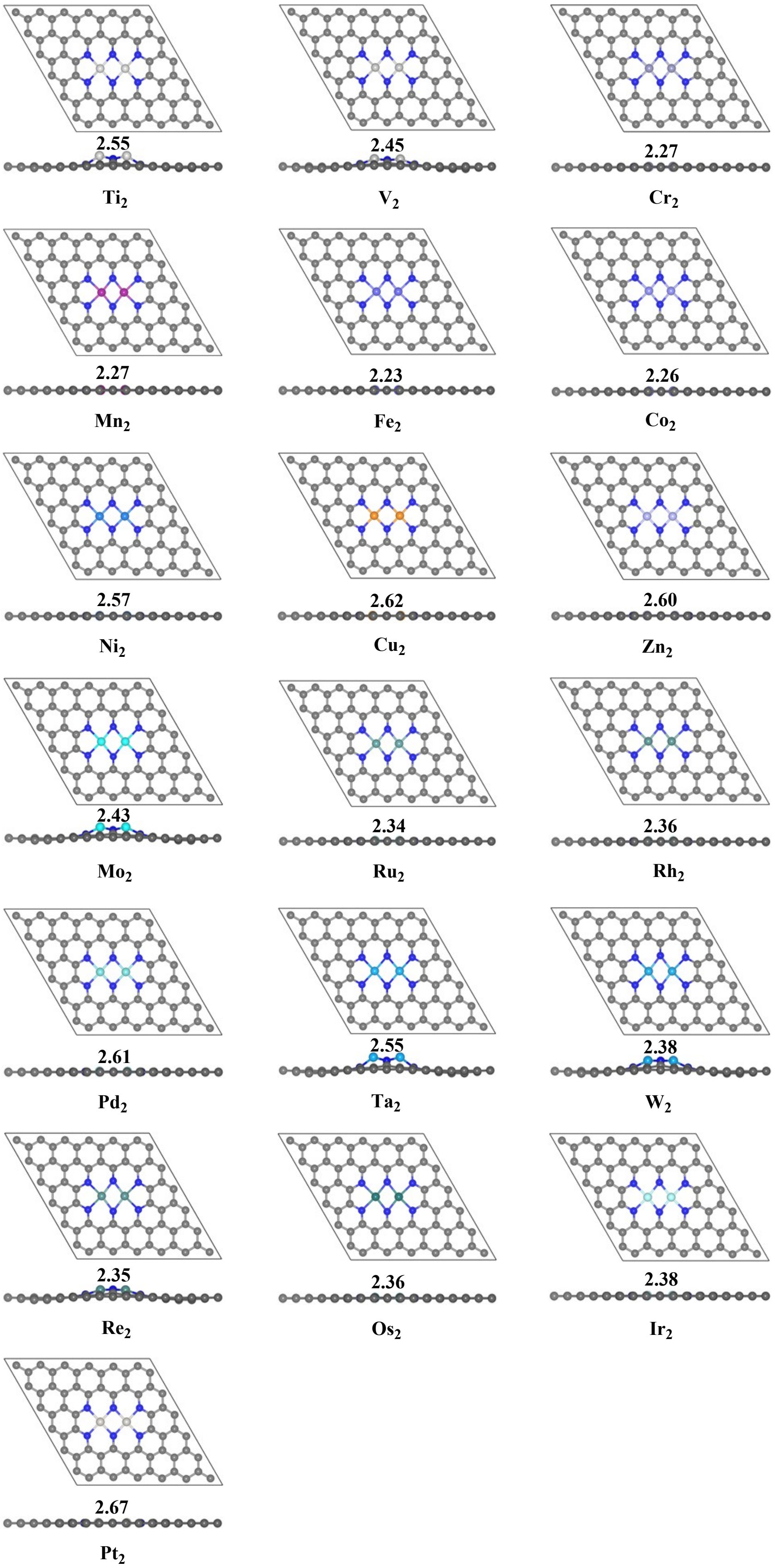

Fig. S1 Top and side views of various homonuclear TM_2N_6@graphene DMACs with distances between two metal atoms in angstroms (Å).

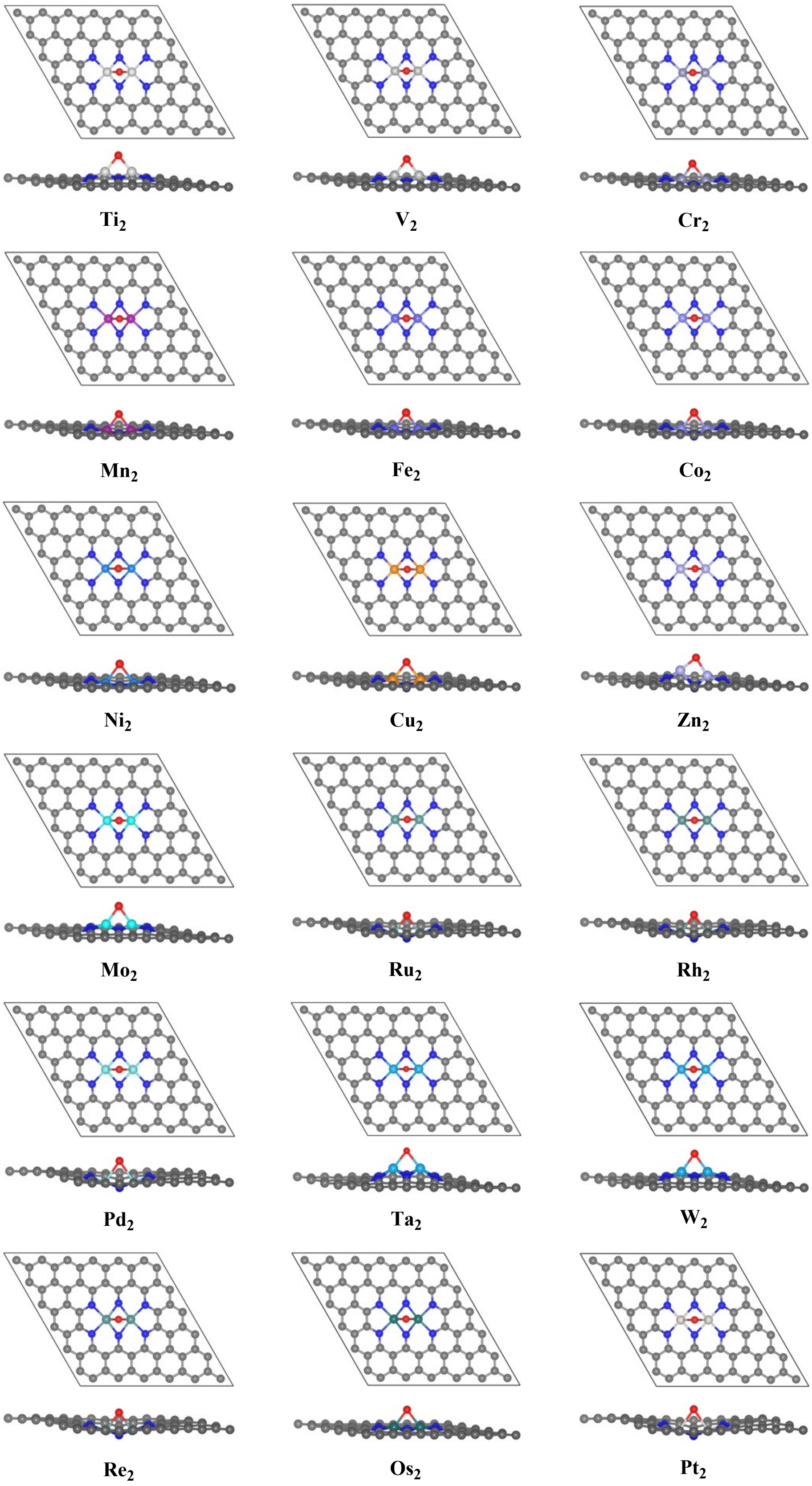

Fig. S2 Top and side views of O atom absorbed at **site-1** on various TM_2N_6@graphenes.

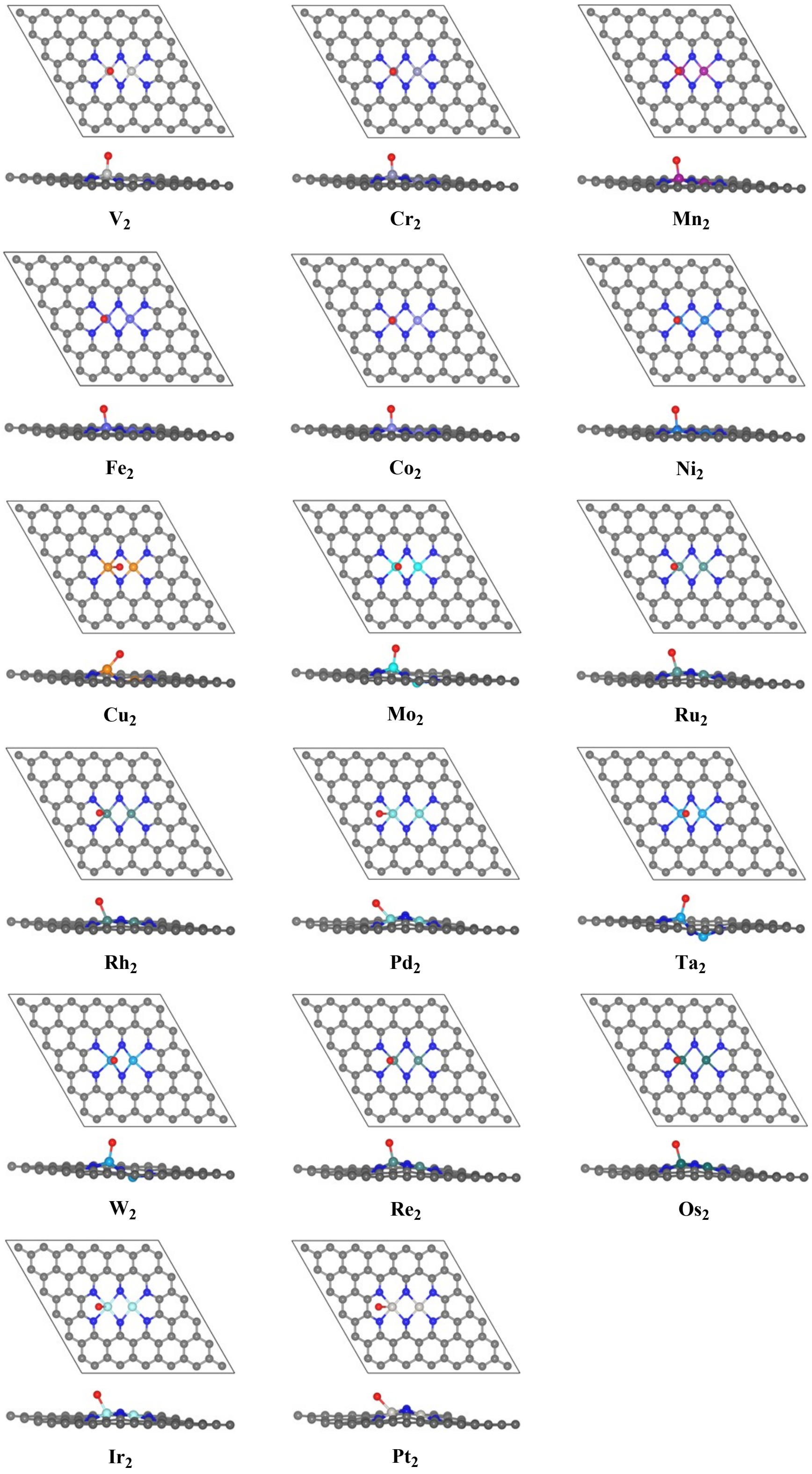

Fig. S3 Top and side views of O atom absorbed at **site-2** on various TM_2N_6@graphenes.

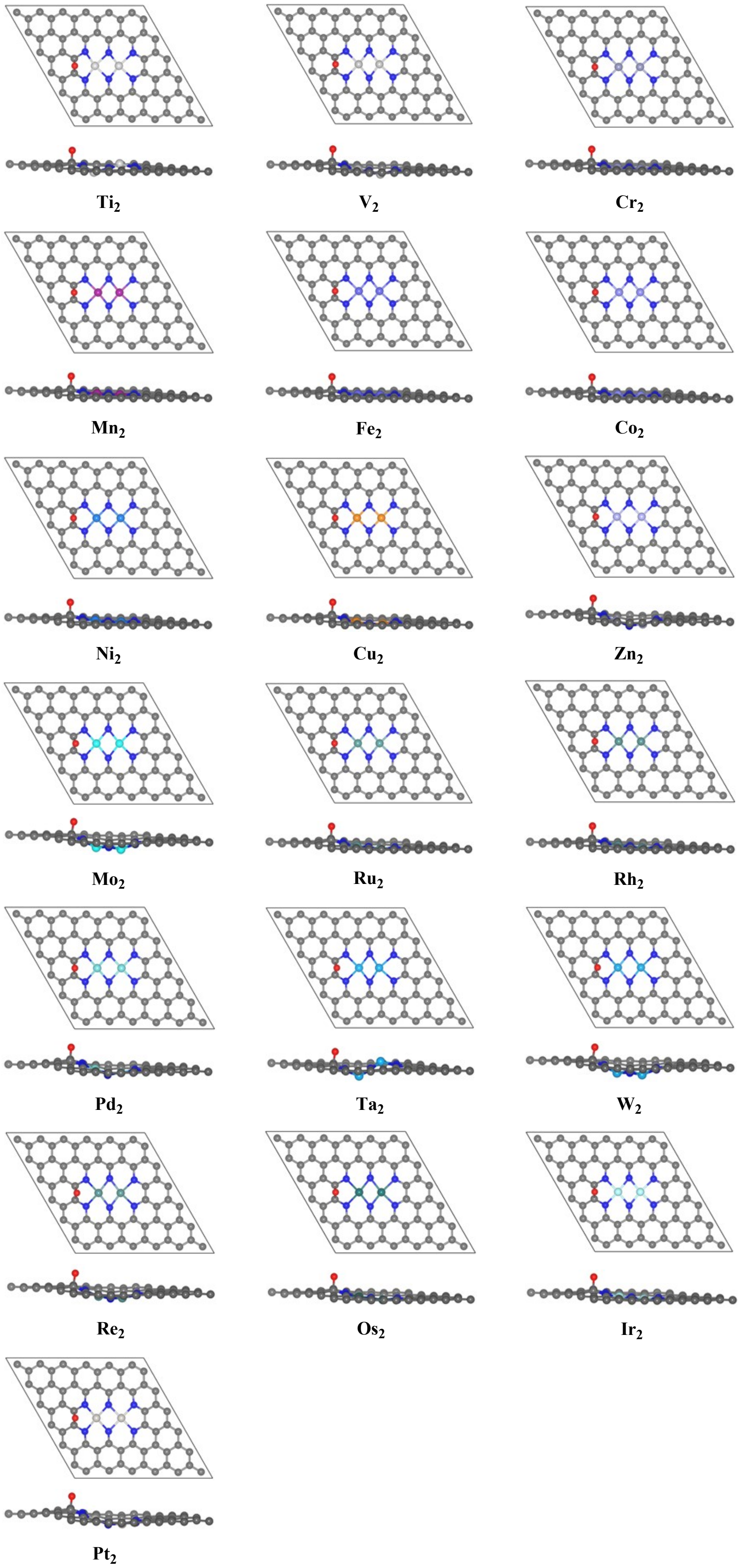

Fig. S4 Top and side views of O atom absorbed at **site-3** on various TM_2N_6@graphenes.

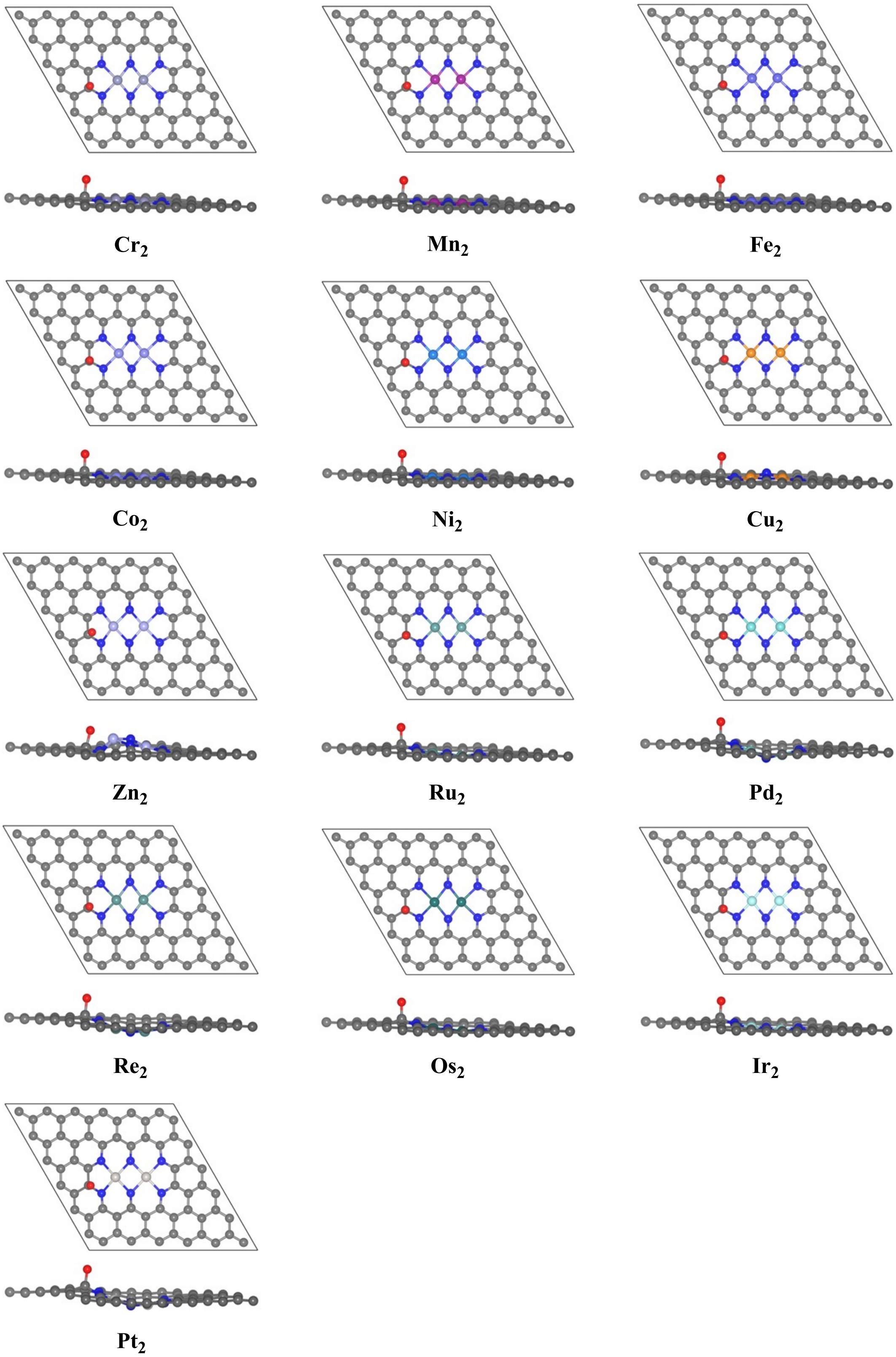

Fig. S5 Top and side views of O atom absorbed at **site-4** on various TM_2N_6@graphenes.

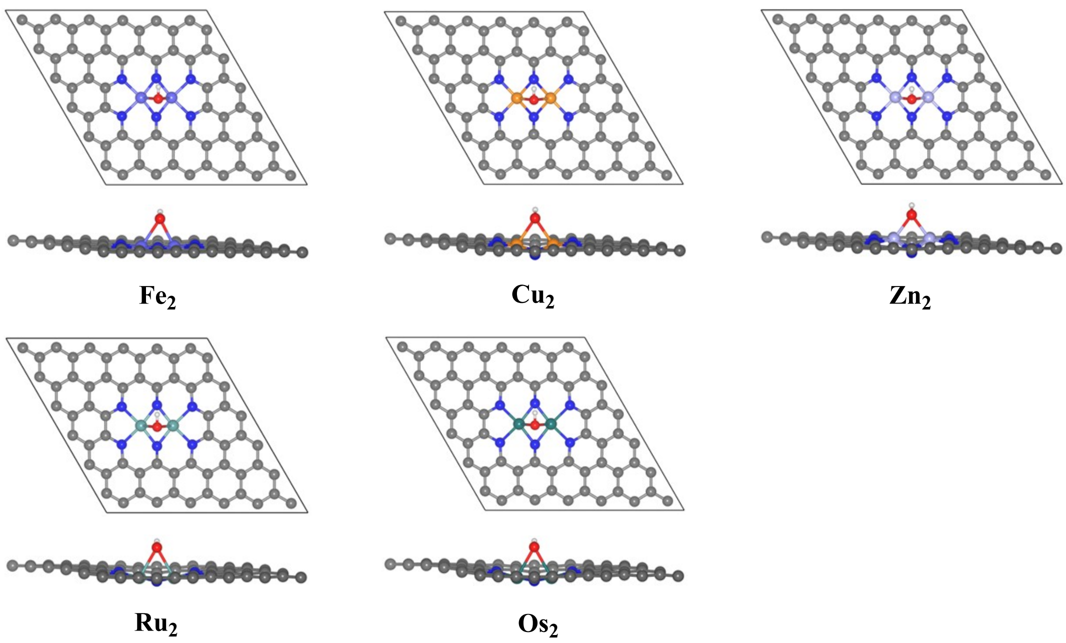

Fig. S6 Top and side views of OH absorbed at **site-1** on various TM_2N_6@graphenes.

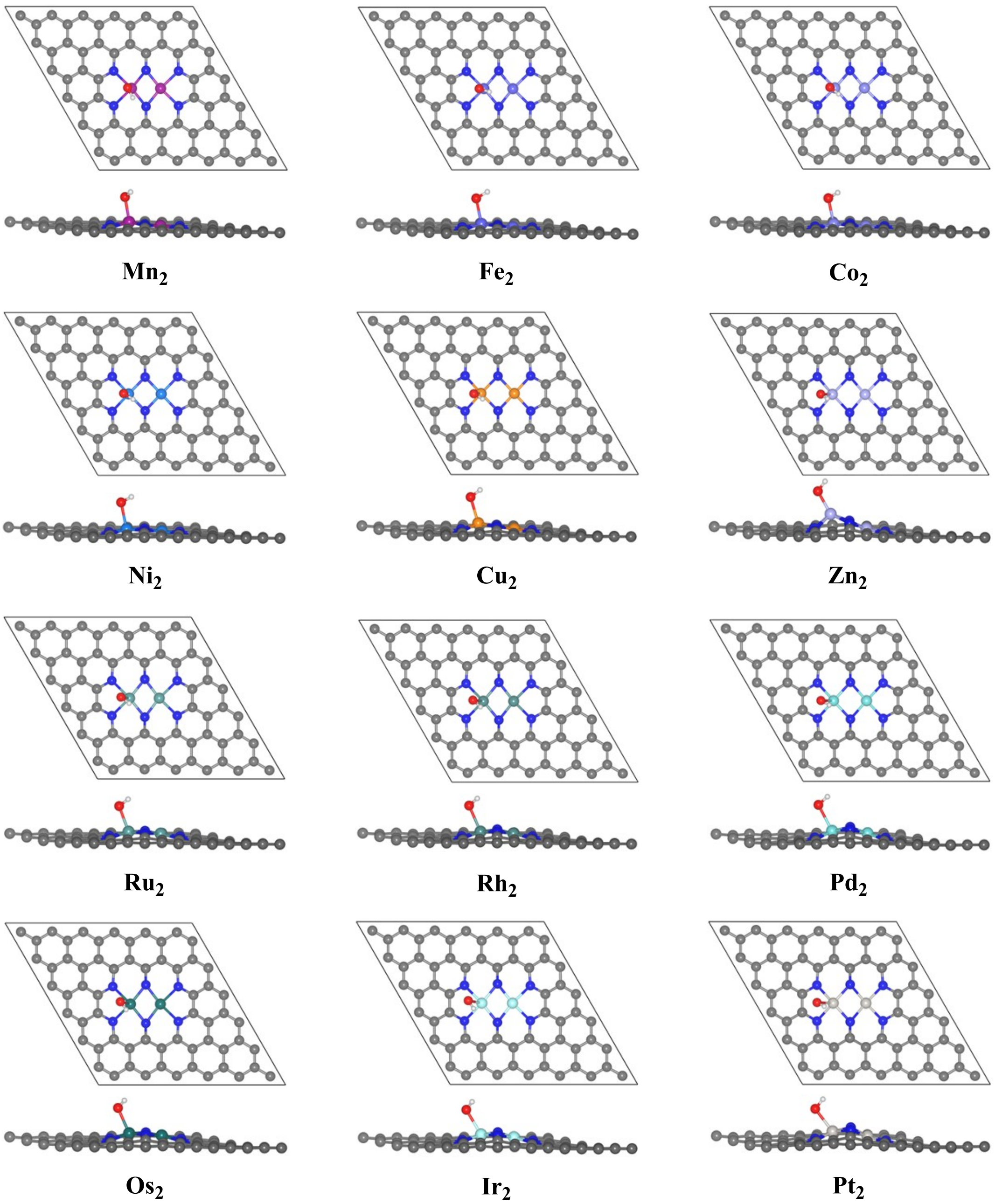

Fig. S7 Top and side views of OH absorbed at **site-2** on various TM_2N_6@graphenes.

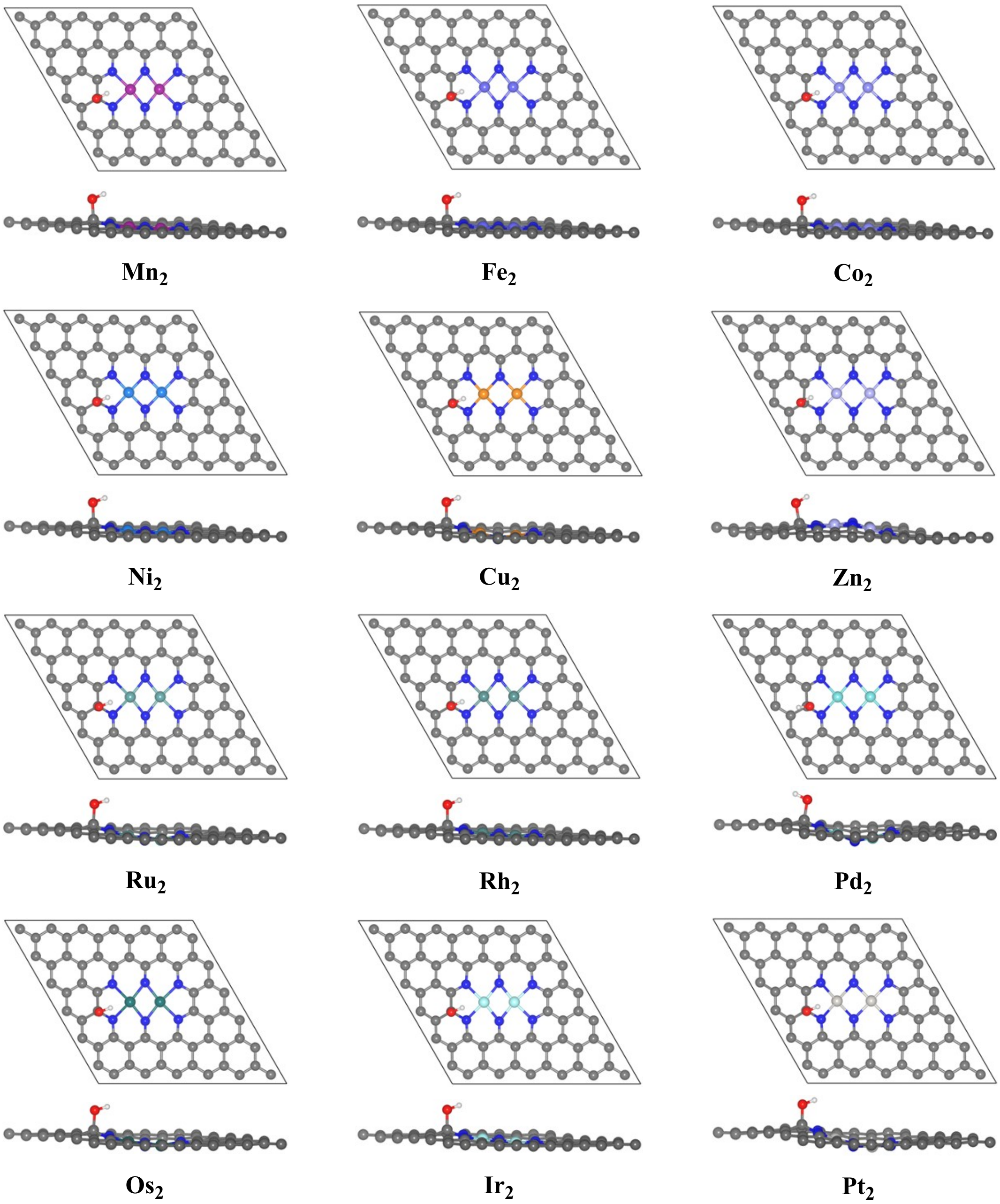

Fig. S8 Top and side views of OH absorbed at **site-4** on various TM_2N_6@graphenes.

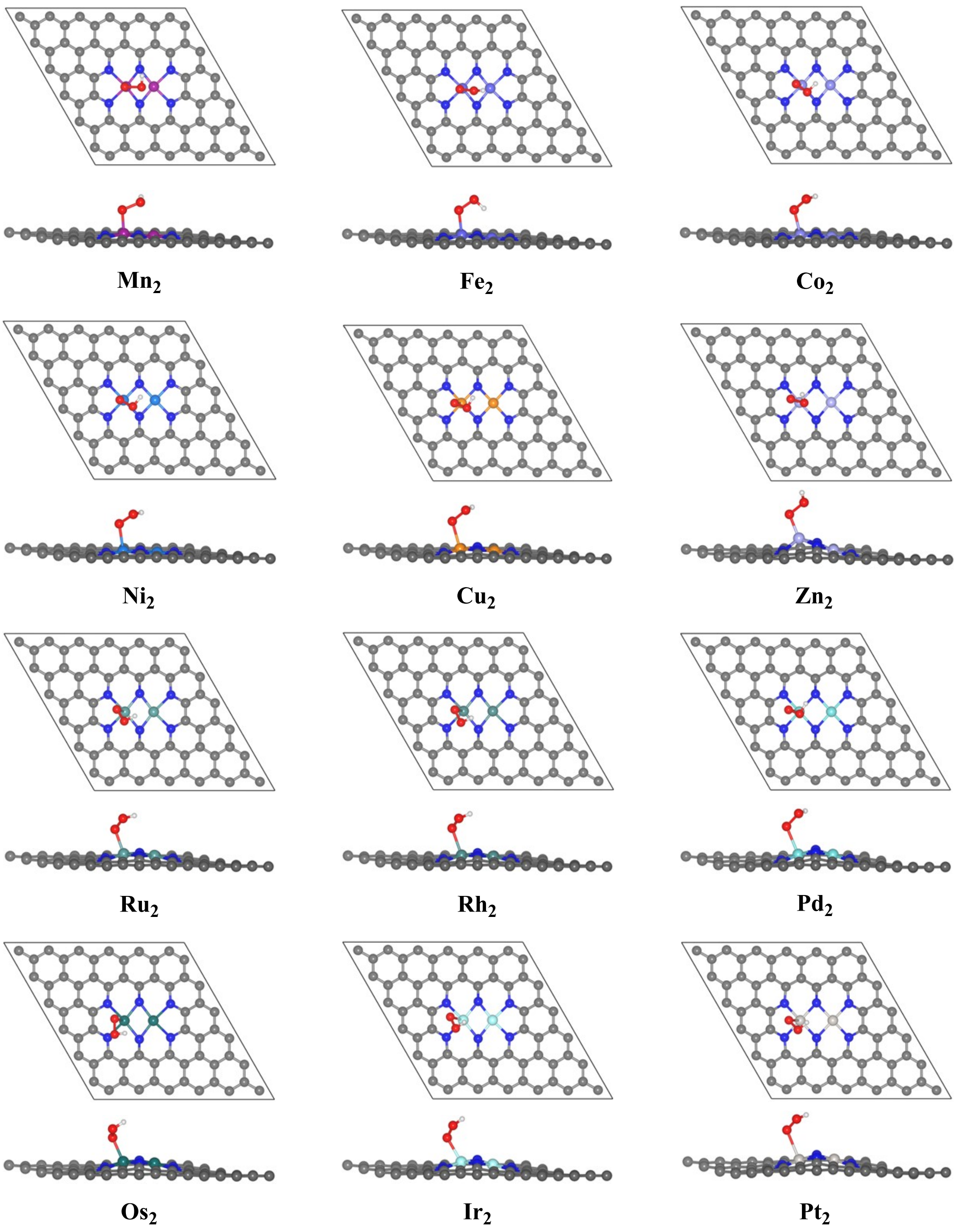

Fig. S9 Top and side views of OOH absorbed on various TM_2N_6@graphenes.

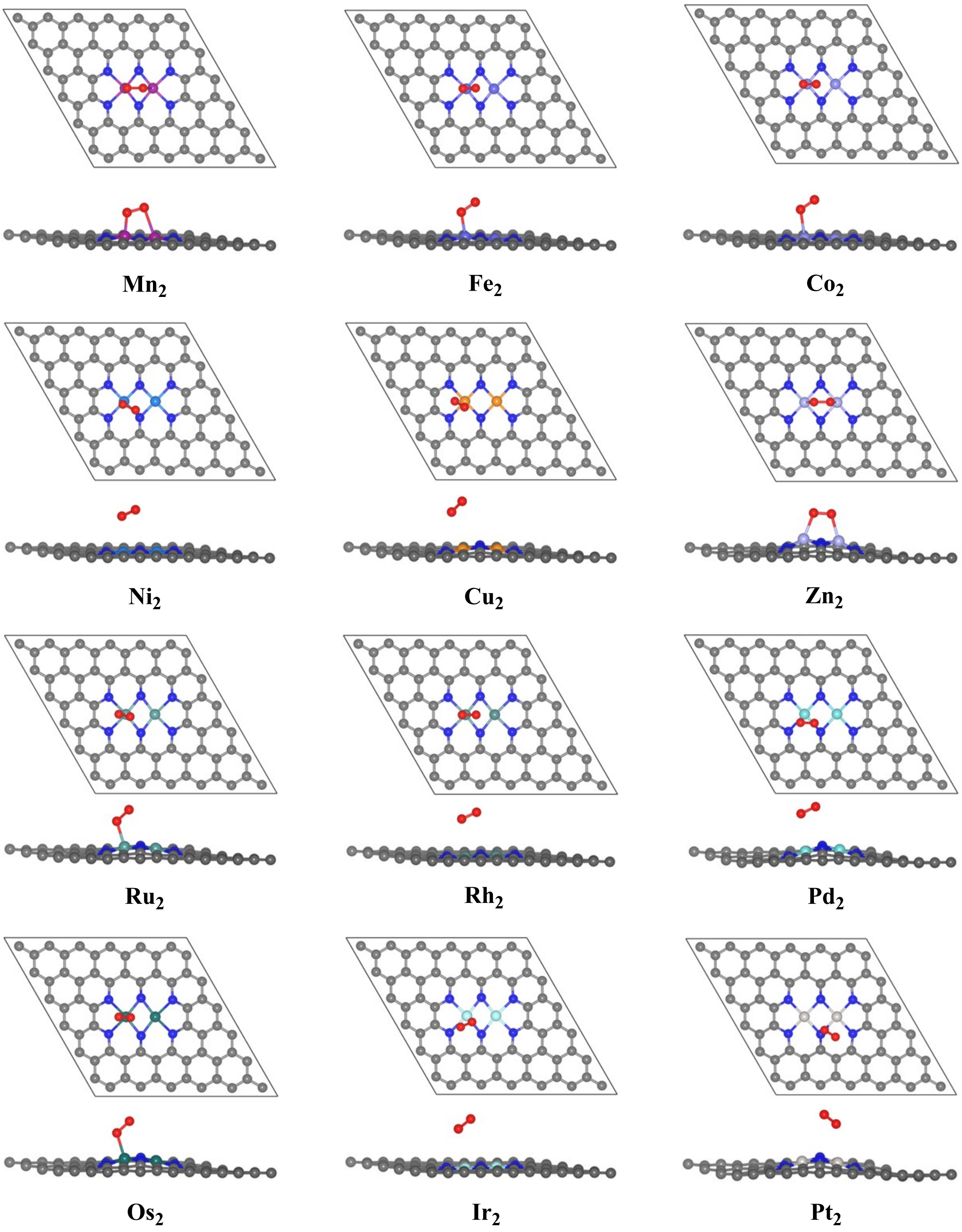

Fig. S10 Top and side views of OO absorbed on various TM_2N_6@graphenes.

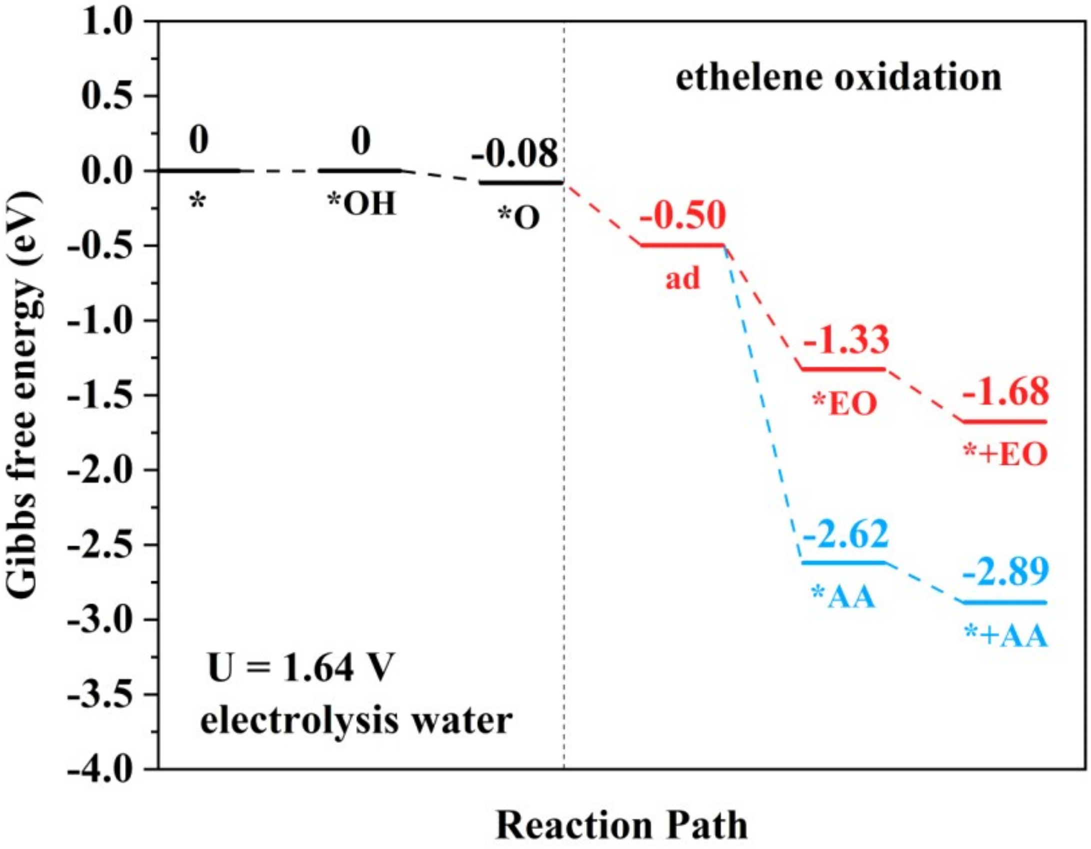

Fig. S11 Gibbs free energy change diagrams for ethylene electro-oxidation on Co_2N_6@graphenes at the lowest applied potential to generate *O intermediates at pH=0.

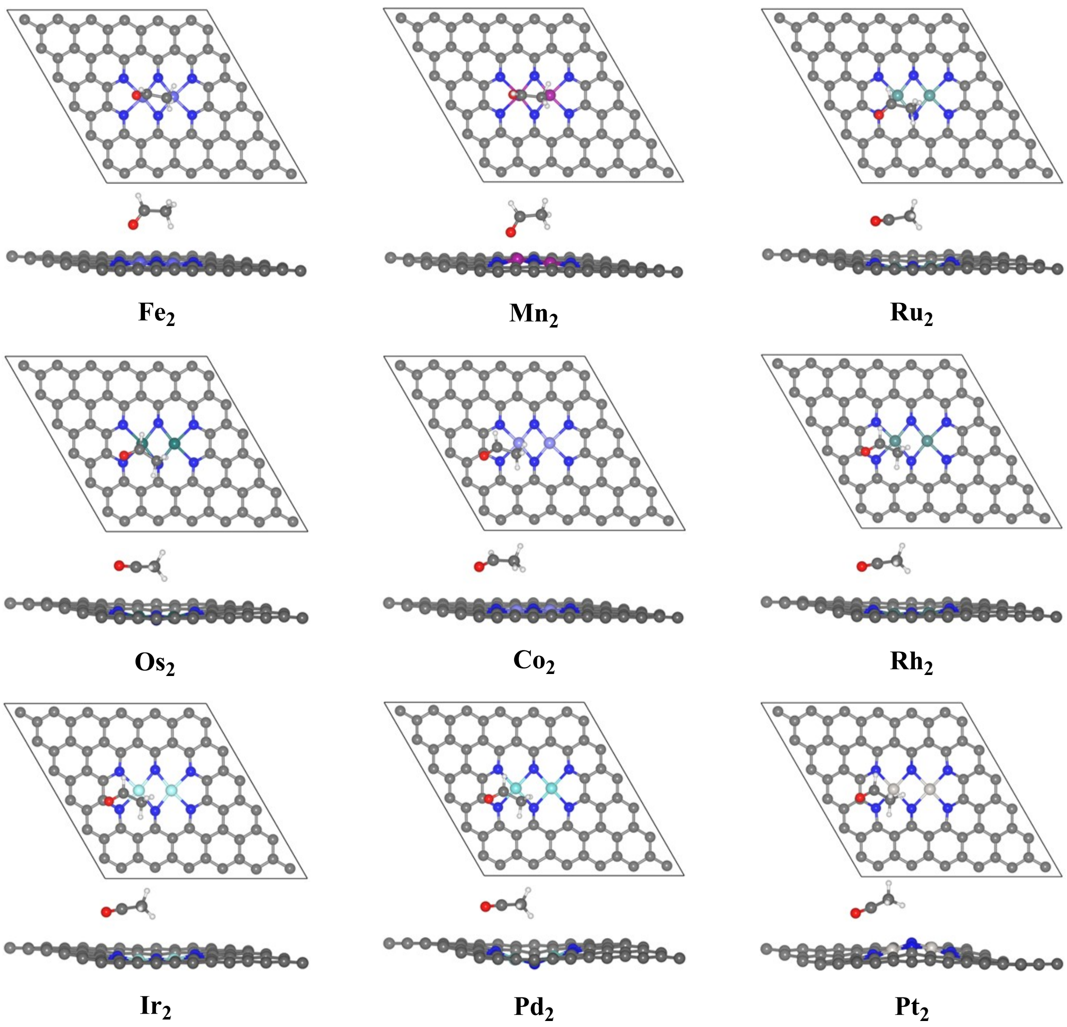

Fig. S12 Top and side views of acetaldehyde absorbed on various TM_2N_6@graphenes.

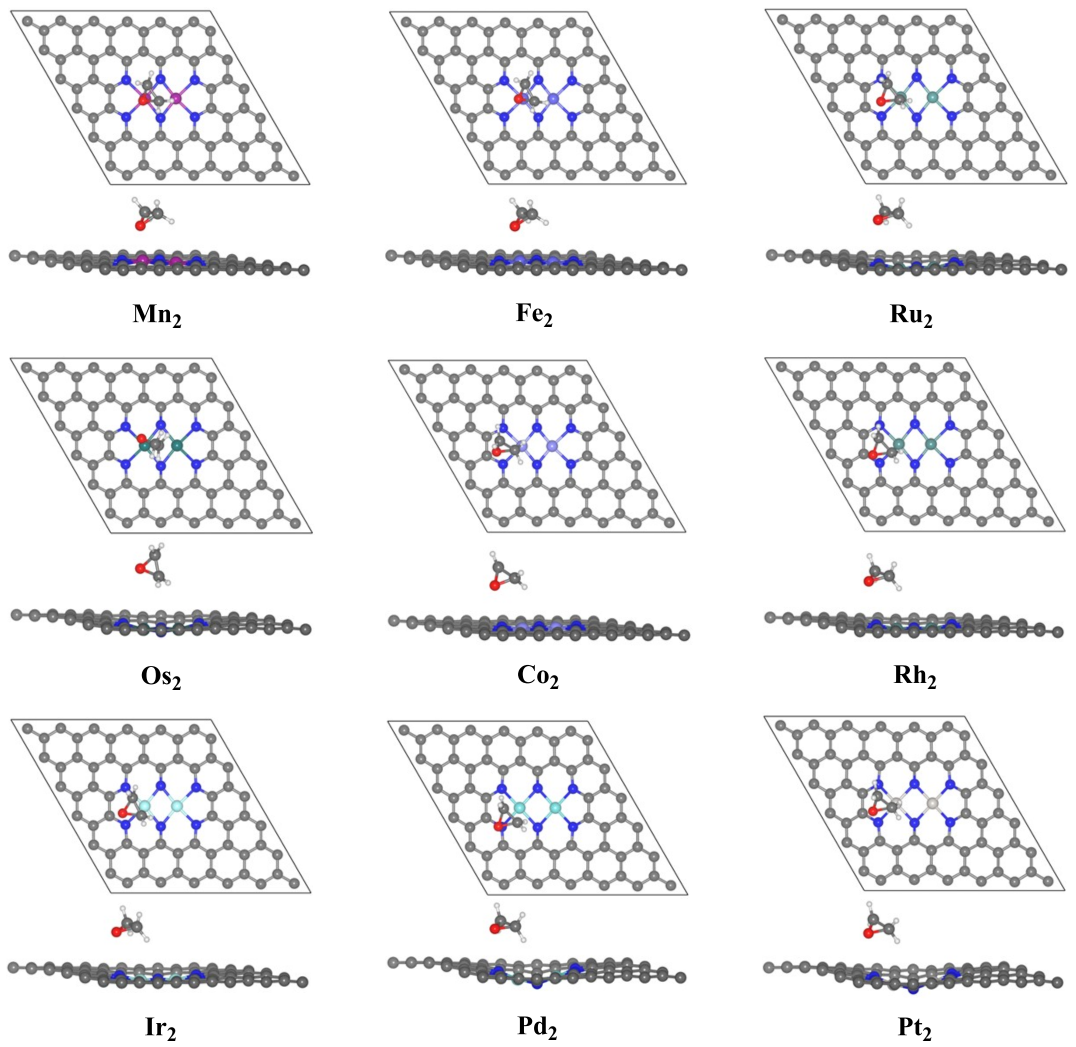

Fig. S13 Top and side views of ethylene oxide absorbed on various TM_2N_6@graphenes.

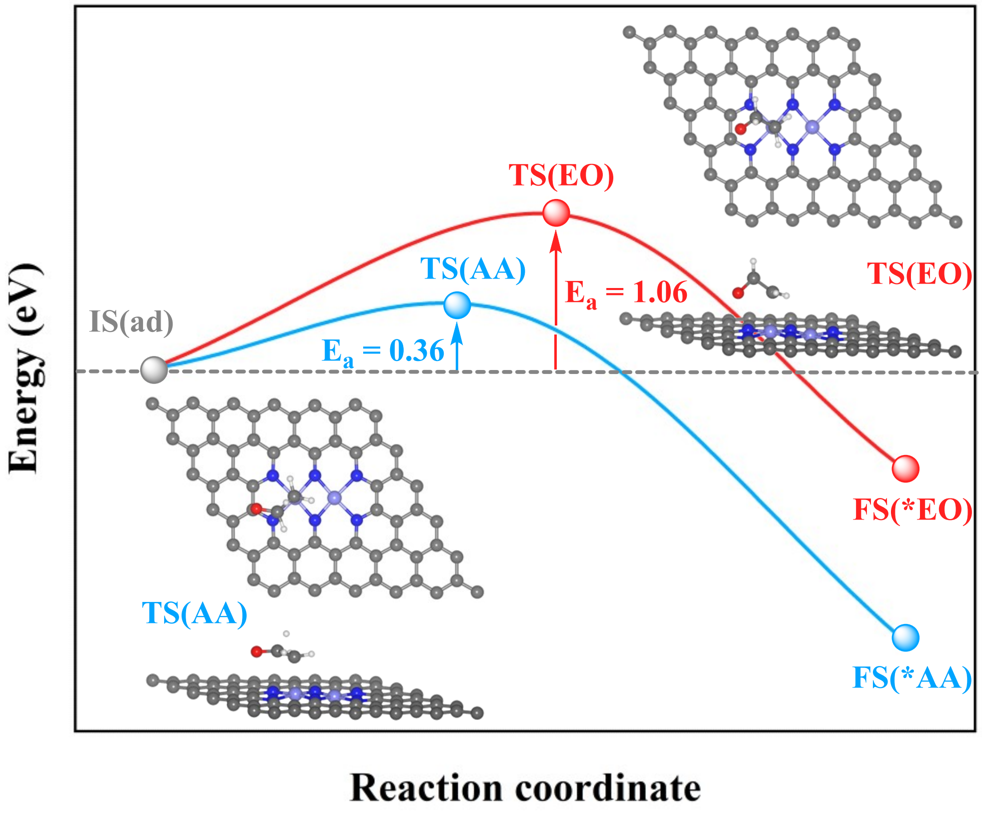

Fig. S14 Kinetic energy barriers and transition state structures of AA and EO generation through C-TM mechanism on Co_2N_6@graphenes.

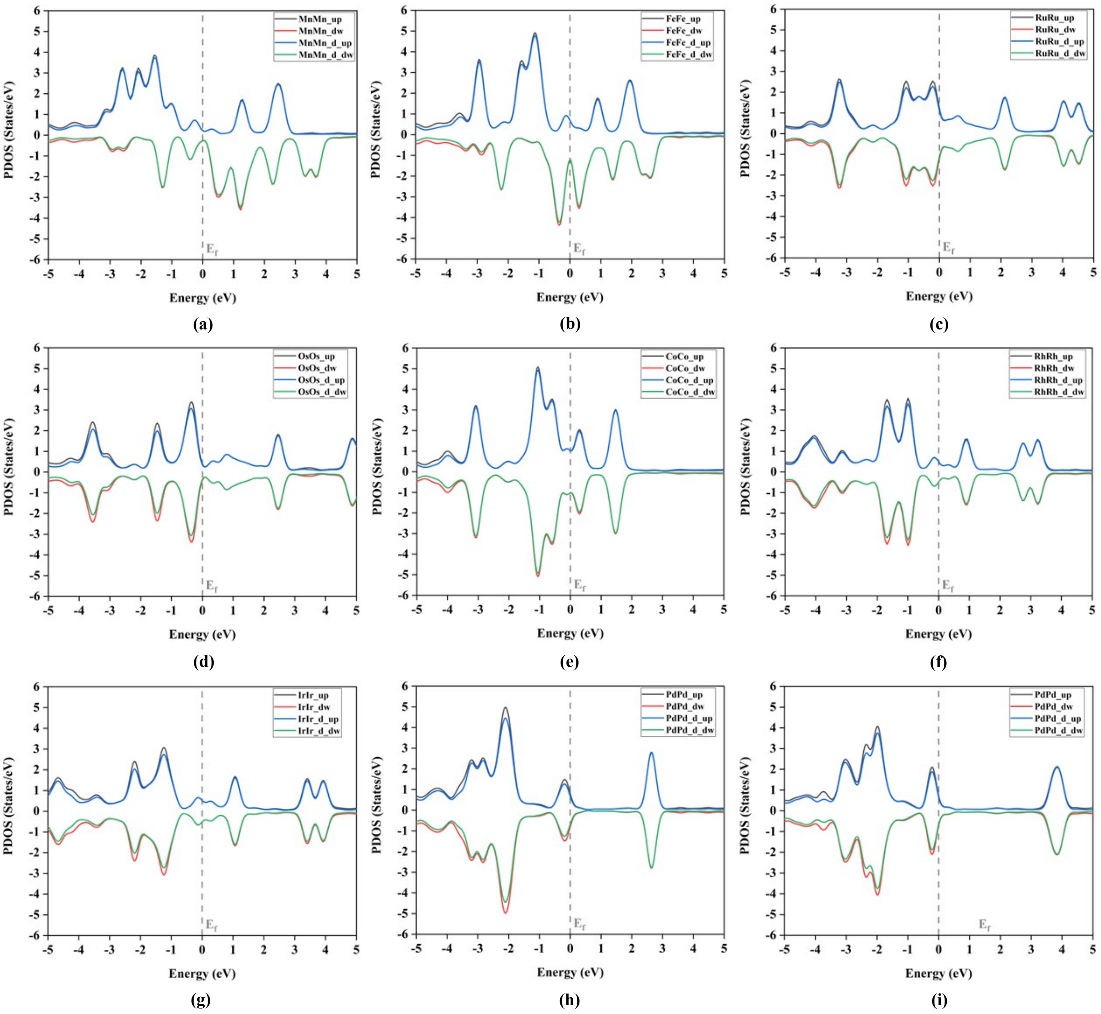

Fig. S15 Projected density of states (PDOS) of the d orbitals of the two metals in TM_2N_6@graphenes.

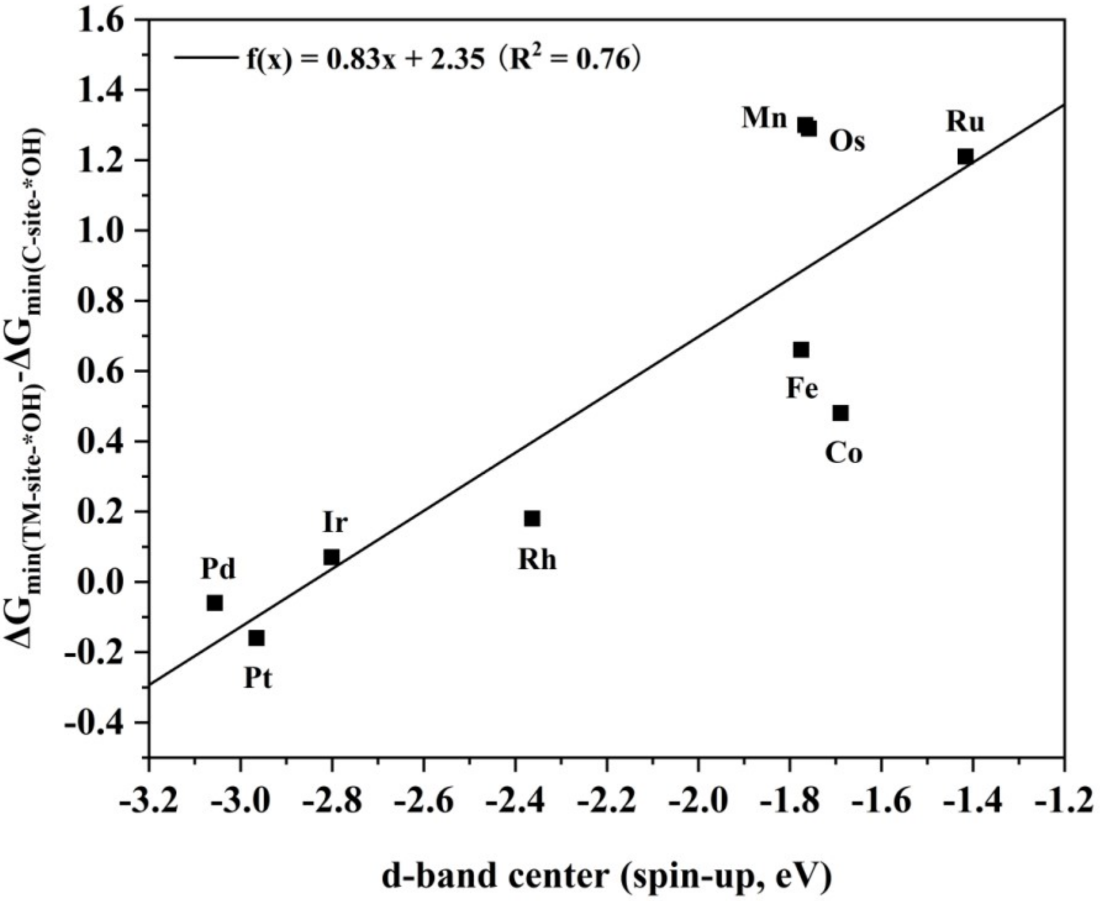

Fig. S16 Linear relationship between d-band center of metals in TM_2N_6@graphenes and energy difference of the most stable OH adsorption sites between metal and C sites.

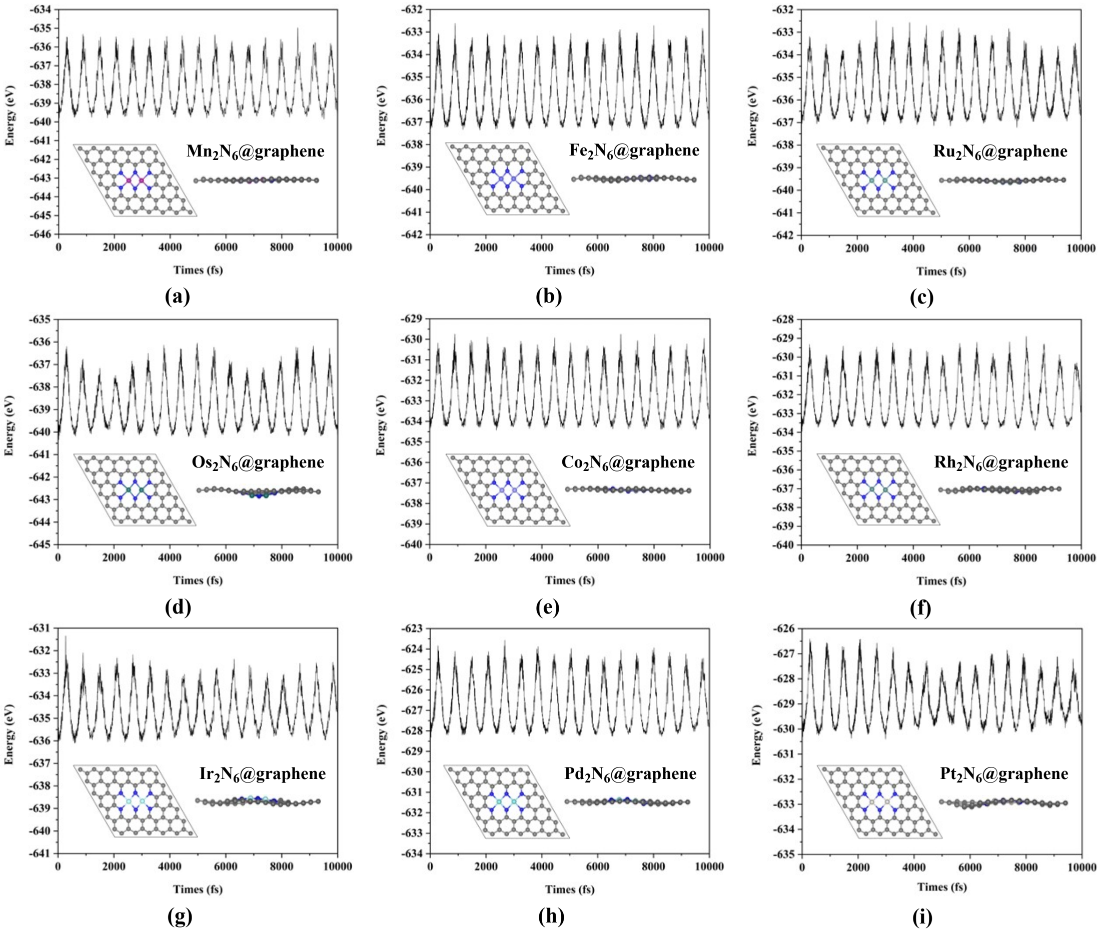

Fig. S17 Evolution of the total energy of 9 TM_2N_6@graphene at 300 K, and the total times duration 10 ps. Snapshots of structures at the end of AIMD simulations are also shown.

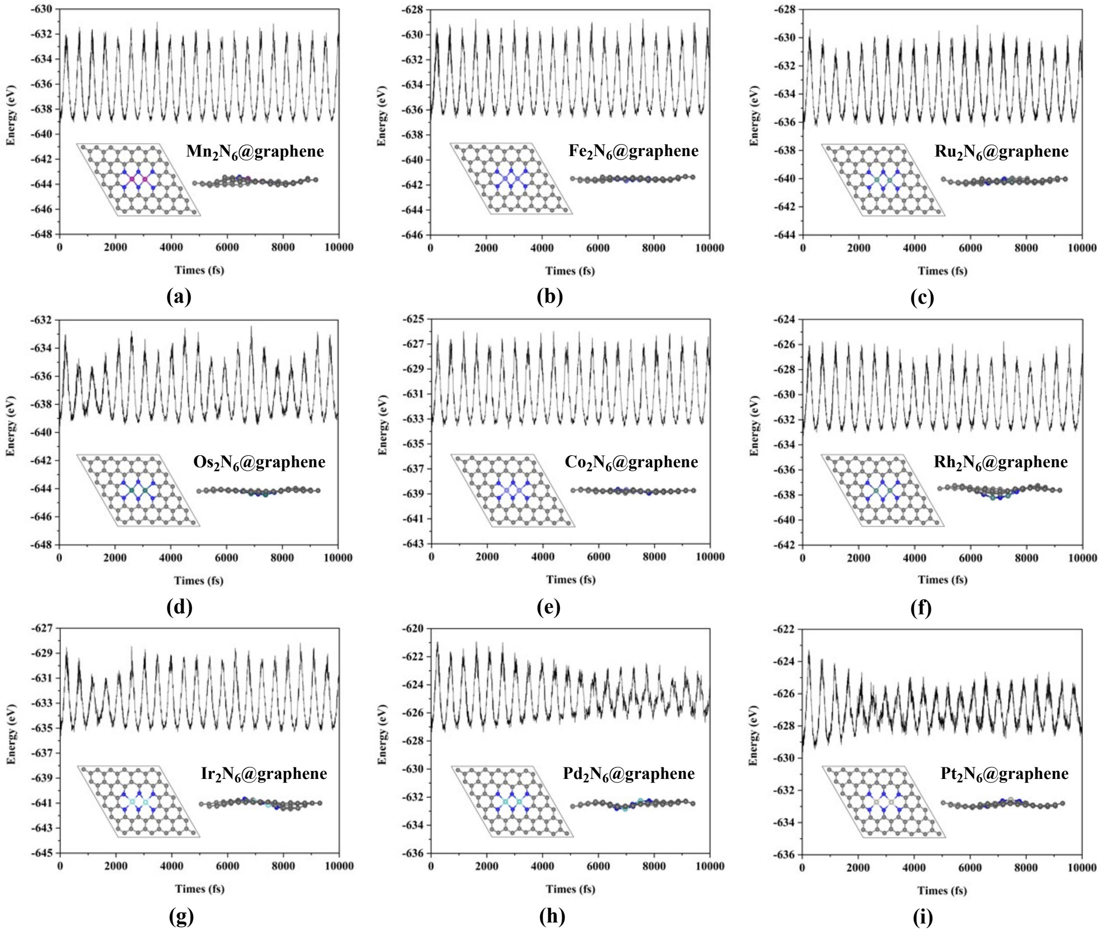

Fig. S18 Evolution of the total energy of 9 TM_2N_6@graphene at 500 K, and the total times duration 10 ps. Snapshots of structures at the end of AIMD simulations are also shown.
